# Mimicking the Electron Transport Chain and Active Site of [FeFe] Hydrogenases in One Metal–Organic Framework: Factors That Influence Charge Transport

**DOI:** 10.1021/jacs.1c01361

**Published:** 2021-05-24

**Authors:** Ashleigh T. Castner, Ben A. Johnson, Seth M. Cohen, Sascha Ott

**Affiliations:** † Department of Chemistry - Ångström Laboratory, Uppsala University, Box 523, 75120 Uppsala, Sweden; ‡ Department of Chemistry and Biochemistry, University of California San Diego, La Jolla, California 92023-0358, United States

## Abstract

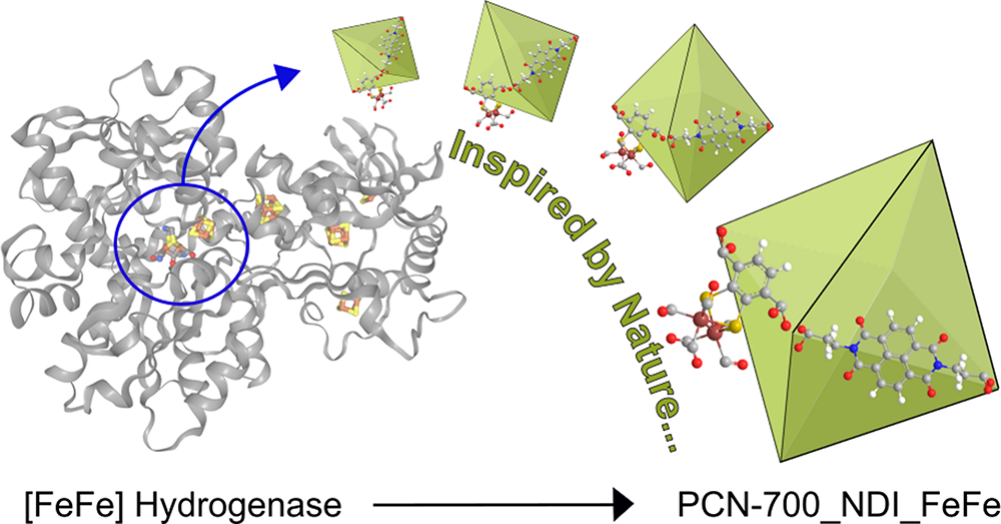

[FeFe] hydrogenase (H_2_ase) enzymes are effective proton reduction catalysts capable of forming molecular dihydrogen with a high turnover frequency at low overpotential. The active sites of these enzymes are buried within the protein structures, and substrates required for hydrogen evolution (both protons and electrons) are shuttled to the active sites through channels from the protein surface. Metal–organic frameworks (MOFs) provide a unique platform for mimicking such enzymes due to their inherent porosity which permits substrate diffusion and their structural tunability which allows for the incorporation of multiple functional linkers. Herein, we describe the preparation and characterization of a redox-active PCN-700-based MOF (PCN = porous coordination network) that features both a biomimetic model of the [FeFe] H_2_ase active site as well as a redox-active linker that acts as an electron mediator, thereby mimicking the function of [4Fe4S] clusters in the enzyme. Rigorous studies on the dual-functionalized MOF by cyclic voltammetry (CV) reveal similarities to the natural system but also important limitations in the MOF-enzyme analogy. Most importantly, and in contrast to the enzyme, restrictions apply to the total concentration of reduced linkers and charge-balancing counter cations that can be accommodated within the MOF. Successive charging of the MOF results in nonideal interactions between linkers and restricted mobility of charge-compensating redox-inactive counterions. Consequently, apparent diffusion coefficients are no longer constant, and expected redox features in the CVs of the materials are absent. Such nonlinear effects may play an important role in MOFs for (electro)catalytic applications.

## Introduction

**Figure 1:**
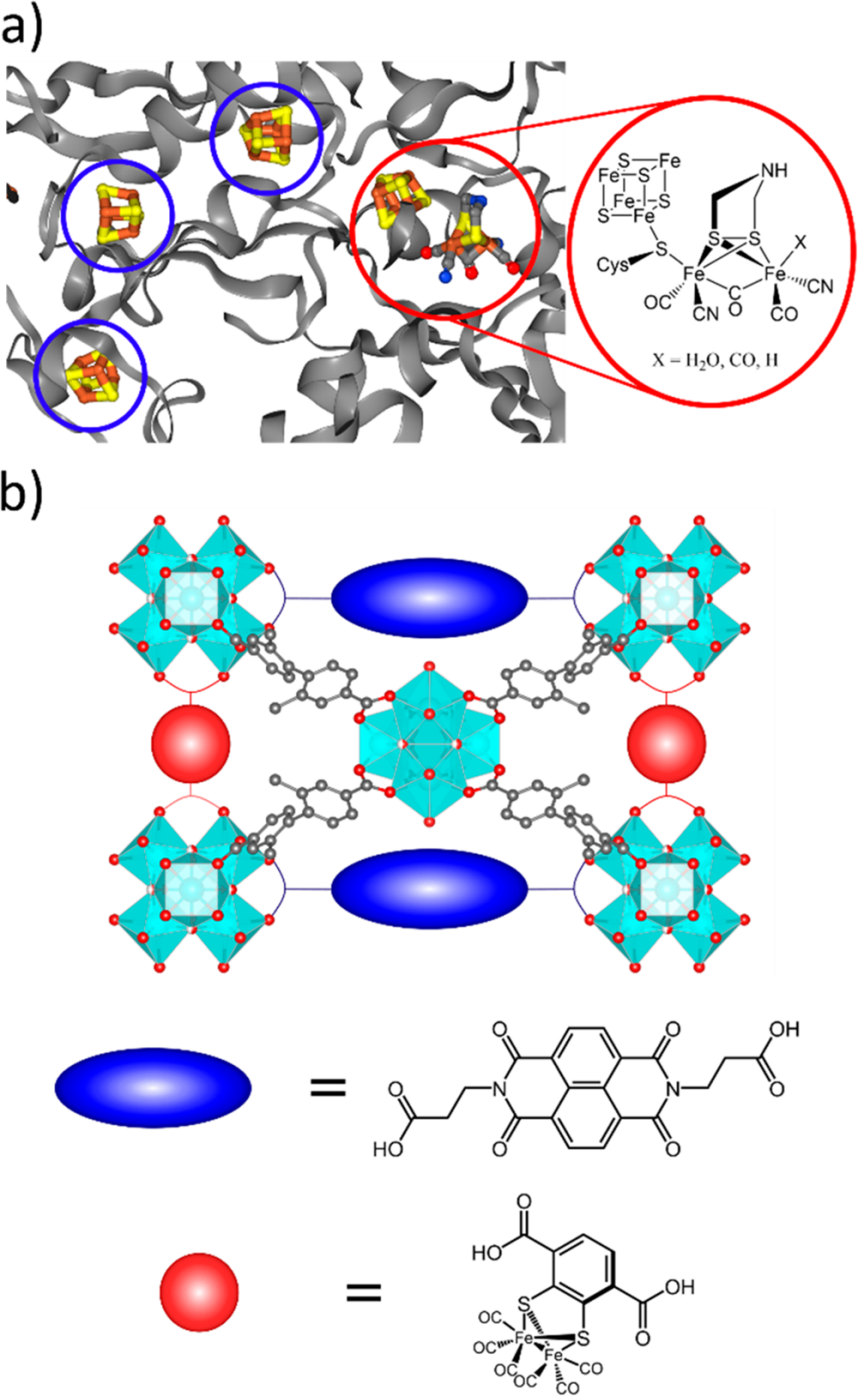
(a) Crystal structure of [FeFe] hydrogenase (*Clostridium pasteurianum* CpI; PDB: 4XDC)^31^ active site pocket and representation of catalytic active site. (b) Representation of biomimetic PCN-700_NDI_FeFe MOF in scaffold MOF PCN-700 (CCDC deposition number: 1036874).^28^ The blue and red circles in parts a and b highlight the enzymatic features ([4Fe4S] clusters and active site) and their corresponding model NDI and Fe_2_ linkers. Hydrogenase structure created with NGL Viewer.^32^

Hydrogenase enzymes are Nature’s catalysts for the interconversion of hydrogen, protons, and electrons. In particular, the [FeFe] hydrogenases (H_2_ases) have attracted significant attention from the bioinorganic modeling community ever since the report of the first enzyme crystal structure more than 20 years ago.^1,2^ This interest is motivated by the fact that [FeFe] H_2_ases are the most active for hydrogen evolution, and mimicking their performance in a synthetic system would allow for the large scale production of hydrogen based on earth-abundant metals. As the name indicates, the [FeFe] H_2_ase active site is composed of two Fe centers that are connected by a bridging dithiolato cofactor and ligated by CO and CN^–^ ligands (Figure 1a). One of the Fe centers at the Fe_2_ subsite is coordinated by an additional thiolate from a nearby cysteine residue, which in turn is connected to a [4Fe4S] cluster. This cluster is the first of as many as four FeS clusters that provides the transport pathway for electrons to access the Fe_2_ subsite, which is deeply buried within the enzyme for protection.^3^ A significant amount of work has focused on preparing structural and functional models of the Fe_2_ subsite of the [FeFe] H_2_ases,^4−6^ with less than a handful of reports also taking the [4Fe4S] cluster into consideration.^7,8^ From spectroscopic and theoretical studies of the enzyme active site, it is clear that the two units, also referred to as the H-cluster, are a single, strongly coupled electronic system.^9,10^

Considering their potential importance in future energy schemes, models of the Fe_2_ subsite have been incorporated in synthetic supports such as polymers,^11^ dendrimers,^12^ or metal–organic frameworks (MOFs).^13,14^ Despite clearly not being biological in nature, the latter have functional similarities to enzymes.^15^ MOFs are highly porous, crystalline materials that are composed of polydentate organic linkers and inorganic nodes known as secondary binding units (SBUs). Like enzymes, MOFs feature well-defined channels for substrate and product transport. Molecular units that are catalytically active can be spatially isolated and protected from diffusional encounters with each other. Because they can be prepared from a broad range of different nodes and linkers, MOFs are highly tunable. In particular, modifications in the vicinity of active sites by functionalization of organic linkers can fine-tune the local chemical environment around the catalysts, which is an essential feature of enzymes.

The use of MOFs as scaffolds for electrocatalytic applications is generally impeded by the intrinsic insulating nature of MOFs. Among other strategies,^16^ it has been shown in recent years that this shortcoming can be overcome by incorporating redox-active linkers in the MOFs that promote electron transport through a hopping mechanism.^17^ This process is described as a diffusional electron transfer between immobilized redox-active linkers. If the redox-active linkers are at the same time catalytically active, a new class of MOF-based electrocatalysts can be envisaged.^18^ This has been realized in recent years, with the most prominent examples of this strategy being those composed of porphyrin linkers,^19−21^ and the replacement of biphenyldicarboxylates (bpdc) by bipyridine (bpy) linkers which then host a variety of different transition metal fragments.^22−24^ More recently, the intentional design of new MOFs in which every linker is a redox catalyst has been described.^25^

In all of these reports, the redox-active metallo-linkers are both redox mediators and catalytic sites,^19−25^ a situation that is inefficient as the two roles cannot be optimized individually and can lead to kinetic limitations. The decoupling of the two functions has precedence in polymer-coated electrodes for electrocatalysis applications.^26,27^ In Nature, the charge transport function is also separated from the catalytic function, as illustrated in the [FeFe] H_2_ase.

Herein, we mimic this design for the first time in a MOF. The function of the [4Fe4S]-based enzymatic electron transport chain is modeled by an organic redox-active naphthalene diimide-based (NDI) linker, while the Fe_2_ subsite is modeled by a structurally related [FeFe](dcbdt)(CO)_6_ (dcbdt = 1,4-dicarboxylbenzene-2,3-dithiolate) complex (Figure 1b). The NDI linker and the Fe_2_ complex possess matching formal potentials for energy efficient electron transfer reactions. Moreover, the two units reside in predetermined positions within the MOF crystal, taking advantage of a postsynthetic linker insertion strategy.^28^ In fact, the NDI-to-Fe_2_ distances in the target PCN-700_NDI_FeFe are projected to be in the same range as those between [4Fe4S] clusters in [FeFe] and [NiFe] H_2_ases and also between [4Fe4S] clusters and the redox-active Ni site in the latter H_2_ase (see Figure S8).^3,29^ Through detailed voltammetric investigations, the interplay between the two functional linkers is delineated, and unusual, nonlinear charge transport phenomena are revealed.

## Results and Discussion

### Synthesis and Characterization

In 2015, Zhou and co-workers reported the preparation of the Zr-based MOF PCN-700 (PCN = porous coordination network) which is composed of dimethyl diphenyl dicarboxylate (Me_2_dpdc) linkers and Zr_6_O_4_(OH)_8_(H_2_O)_4_ SBUs.^28^ The components of PCN-700 are similar to those of the canonical Zr-MOF UiO-67, but the presence of methyl groups on the 2- and 2′- positions of the biphenyl linker results in a change to the dihedral angle between the rings, allowing for the formation of MOF crystals with Zr_6_ clusters that are 8-connected rather than the fully 12-connected configuration that is observed for the UiO series of Zr-MOFs. This reduced SBU connectivity leaves four linker vacant pockets that can be postsynthetically filled by linkers of different length (two pockets of 7.0 and 16.4 Å each) via sequential linker installation (SLI).^30^

**Figure 2:**
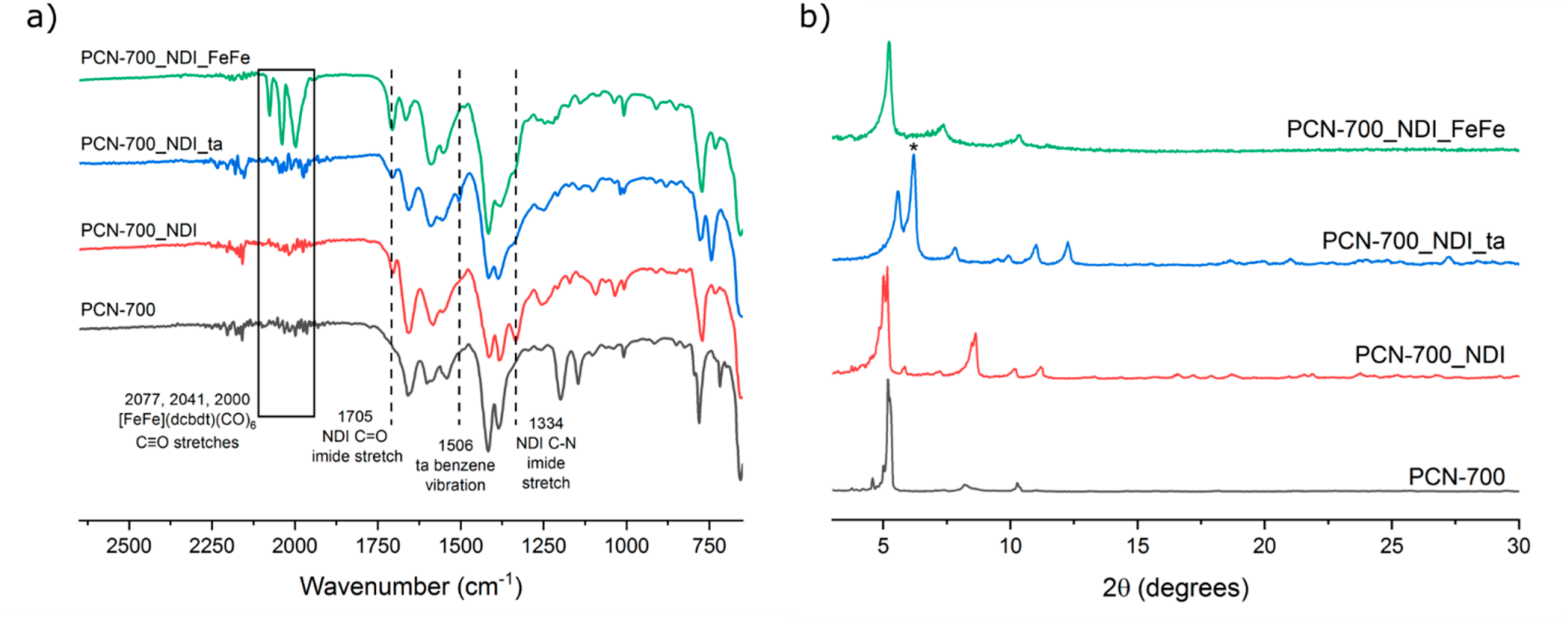
Characterization of PCN-700 materials by (a) ATR-FTIR spectroscopy with diagnostic absorptions of the introduced linkers highlighted and (b) powder X-ray diffraction. The * peak at 2θ around 6.2 denotes a UiO-type phase which is isoreticular to UiO-67 that has also been observed in the original report.^28^ Peaks around this angle also observed in PCN-700 MOFs after linker insertion for different linkers of various length in a subsequent report.^30^

In the present work, the Me_2_dpdc linker was obtained by first preparing the boronic acid ester of the appropriate methyl benzoate. The symmetric methyl ester protected linker was then obtained via a Pd-catalyzed Suzuki–Miyauru coupling and the final dicarboxylate linker generated via base cleavage of the methyl ester protecting group (see the Supporting Information for details, Figures S1 and S2). [FeFe](dcbdt)(CO)_6_ and PCN-700 were prepared as previously reported.^13,28^ Briefly, Me_2_dpdc and ZrCl_4_ were suspended in DMF with trifluoroacetic acid as a modulator and allowed to react under solvothermal conditions (120 °C, 72 h) to generate the crystalline MOF material, as confirmed by PXRD analysis (Figure 2b).

In the first report of SLI into PCN-700, the large and small pockets were filled by dimethylterphenyl dicarboxylate (Me_2_tpdc) and terephthalic acid (ta), respectively, under elevated temperature. It was found that the smaller ta linker had to be installed first to slightly elongate the larger pocket, thereby allowing for the incorporation of the rigid Me_2_tpdc linker. The opposite SLI order, i.e., first Me_2_tpdc followed by ta insertion, did not result in the desired MOF, as the incorporation of the former led to a significant increase of the second smaller pocket that does not promote bidendate ta insertion.

Considering the size match between the ta linker and the dcbdt ligand in [FeFe](dcbdt)(CO)_6_, we anticipated that the small pocket may accommodate the Fe_2_ complex, while the larger pocket could host an NDI-based linker. As [FeFe](dcbdt)(CO)_6_ is thermally unstable, it would be desirable to introduce it last and under ambient conditions, both of which were not possible in the original report. We hypothesized that both of these features could potentially be implemented into the sequence by changing from a rigid linker like Me_2_tpdc to one that has more conformational flexibility. An NDI analogue with two *N*-propanoic acid substituents has an approximate length of 15.8 Å in its “stretched out” conformation, which is similar to that of the rigid Me_2_tpdc. It is worthwhile mentioning that linkers containing aliphatic hydrocarbons are very unusual in MOF chemistry, but since the overall topology of PCN-700 is already set, we anticipated that the flexibility of the NDI linker may be tolerated, while also allowing for the SLI of [FeFe](dcbdt)(CO)_6_ as the second linker incorporated, and potentially under ambient temperatures.

With this rationale, the flexible NDI linker was incorporated first. Five equivalents of the NDI linker per desired vacant site was allowed to incorporate into PCN-700 in DMF at 75 °C for 24 h, and the resulting material was thoroughly washed with acetone via Soxhlet extraction to obtain the functionalized PCN-700_NDI. The ^1^H NMR spectrum of digested PCN-700_NDI gives a ratio between the Me_2_dpdc and incorporated NDI linker of approximately 8:1.56, corresponding to an NDI linker incorporation yield of 78% (Figure S10, Table S1). NDI linker incorporation and framework integrity were further verified by ATR-FTIR spectroscopy and PXRD (Figure 2). Upon NDI incorporation, the FTIR spectrum of PCN-700_NDI features peaks at ∼1334 and 1705 cm^–1^ that are not present in PCN-700. These peaks arise from characteristic stretches of the cyclic imide at ∼1334 cm^–1^ (C—N stretch) and 1705 cm^–1^ (C=O stretch) and correlate well with those of the homogeneous NDI reference (Figure S6). NDI incorporation also resulted in a color change of the MOF from white to a pale yellow (Figure S7).

After NDI linker incorporation, ta was introduced into PCN-700_NDI. Using the same conditions as for NDI incorporation (5 eq ta, 75 °C, 24 h) resulted in the removal of a significant amount of the NDI linker from PCN-700_NDI and partial replacement by ta. To determine if more mild conditions could be employed for the second linker incorporation as a benefit for maintaining the structural integrity of the [FeFe] catalyst, 2 equivalents of ta per desired vacant site was mixed with PCN-700_NDI in DMF and allowed to incorporate at room temperature for 24 h. The final material was Soxhlet extracted with acetone to remove any trapped linker. The obtained PCN-700_NDI_ta MOF was found to have a Me_2_dpdc:NDI:ta linker ratio of ∼8:1.52:1.8, by ^1^H NMR analysis of digested samples, indicating minimal loss of the NDI ligand (76% occupancy of vacant sites) and a high incorporation yield of ta (90% occupancy) (Figure S11, Table S1). By ATR-FTIR, the signature NDI peaks are still present at ∼1334 and 1705 cm^–1^, demonstrating that the NDI linker remains incorporated in the MOF. In addition, a diagnostic feature appears in the ATR-FTIR spectrum at 1506 cm^–1^ as a result of ta incorporation. In analogy to a report by Zhou,^30^ a partial transformation to a UiO-like phase can be observed depending upon the structural nature of the linkers installed (Figure 2b), while the BET surface area remains in the expected range (see Figures S15 and S16).

Having established effective conditions for SLI of the NDI linker followed by the smaller ta, the targeted dual-functional biomimetic [FeFe] H_2_ase model MOF was prepared. PCN-700_NDI was mixed with 2 equivalents of the active site mimic [FeFe](dcbdt)(CO)_6_ in deoxygenated H_2_O and stirred at room temperature under an inert atmosphere in the dark for 24 h. The resulting material was then washed two to three times with deoxygenated H_2_O and subsequently washed with deoxygenated acetone until no color remained in the liquid phase. ATR-FTIR analysis confirmed retention of the NDI linker and the integrity of the [FeFe] catalyst by the presence of peaks at 2077, 2041, and 2000 cm^–1^ which represent the C≡O stretches of the Fe_2_ linker (Figure 2a). ICP-OES analysis of the Zr to Fe content indicated that the [FeFe] active site model was present in 65–78% of the total linker vacant sites. Incorporation of [FeFe](dcbdt)(CO)_6_ also results in an additional color change from the pale yellow of PCN-700_NDI to a pale orange for the final dual incorporated MOF (Figure S7). Control experiments with [FeFe](bdt)(CO)_6_ that lacks the carboxylate anchoring groups did not result in any detectable linker insertion (Figure S12).

### Electrochemical Characterization

**Figure 3:**
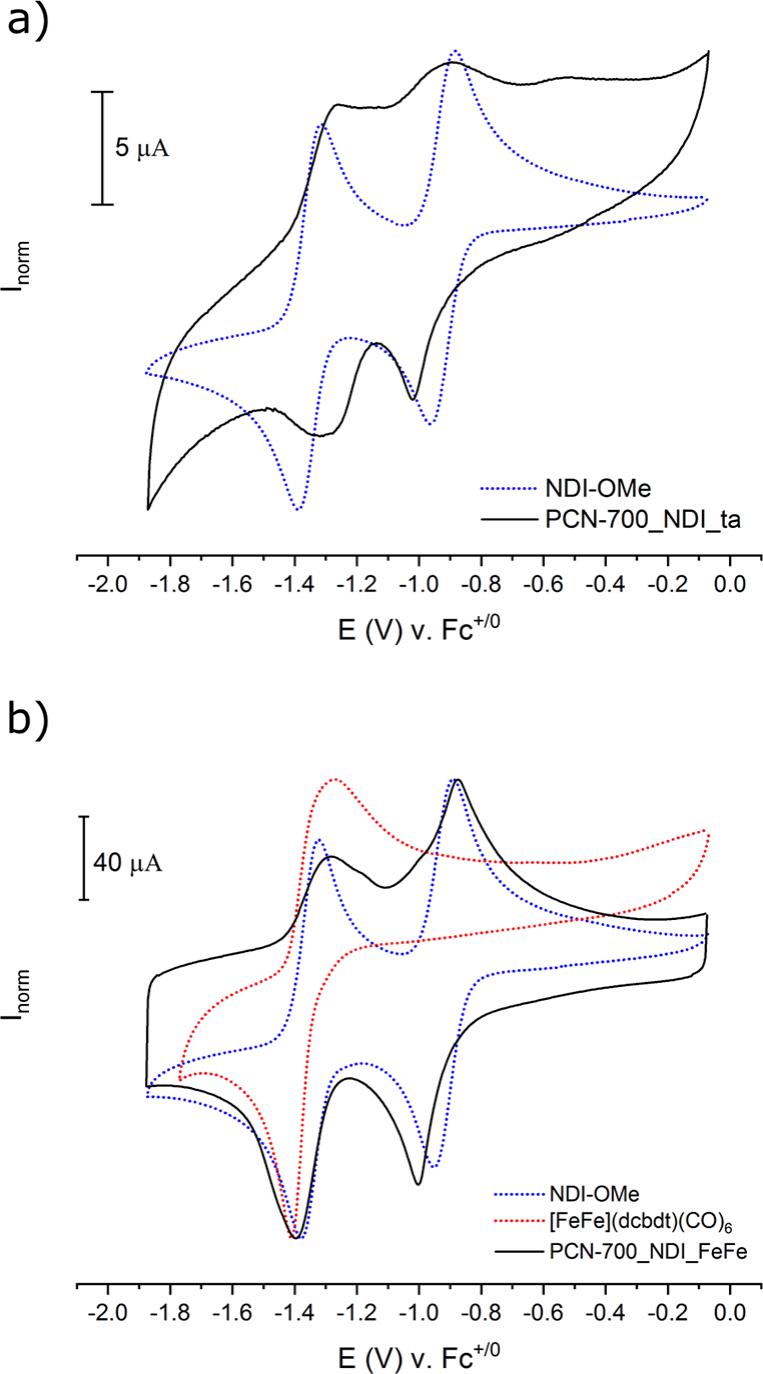
Normalized CVs of homogeneous ligands NDI-OMe (blue) and [FeFe](dcbdt)(CO)_6_ (red) (1 mM) and (a) PCN-700_NDI_ta (black) and (b) PCN-700_NDI_FeFe (black). Indicated currents are for PCN-700_NDI_ta and PCN-700_NDI_FeFe in parts a and b, respectively. Conditions: 0.5 M KPF_6_ in DMF, ν = 50 mV s^–1^.

Electrodes for electrochemical characterization were prepared as described earlier.^33^ In short, the MOF materials were mixed with carbon black and Nafion as a binder to produce a paste that was then dropcast onto glassy carbon substrates. All cyclic voltammograms (CVs) were obtained in DMF with 0.5 M KPF_6_ as supporting electrolyte at a scan rate of 50 mV s^–1^. The CV of PCN-700_NDI_ta (Figure 3a) shows two reversible reductions at *E*_1/2_ = −0.99 and −1.27 V (all potentials are given vs Fc^+/0^). The CV is characterized by a significant capacitive current when compared to the Faradaic waves, reflecting the low proportion of redox-active linkers in PCN-700_NDI_ta (∼12% of the total linkers). The observed reductions are at similar potentials as those of the homogeneous linker (*E*_1/2_ = −0.92 and −1.35 V, Figure 3a, Figure S23) as well as those in other NDI containing MOFs^34^ and are thus assigned to the NDI/NDI^•–^ and NDI^•–^/NDI^2–^ couples, respectively. Noteworthy is the fact that the two reductions in PCN-700_NDI_ta occur within a smaller potential window, though, compared to the situation in the homogeneous linker. As the NDI core is electronically insulated with only aliphatic *N*-substituents, we assign this observed shift in the midpoint potentials to solvation effects within the MOF.

The CV of the dual-functional PCN-700_NDI_FeFe shows two reductions at *E*_1/2_ = −0.94 and −1.34 V (Figure 3b). The redox features in the CV of PCN-700_NDI_FeFe are more distinct than those in PCN-700_NDI_ta, and the CV of the former is characterized by larger Faradaic currents. This is consistent with the presence of a higher amount of redox-active linkers as expected for PCN-700_NDI_FeFe. With ca. 75% of both the large and small pockets hosting the NDI and Fe_2_ linker, respectively, the total percentage of redox addressable linkers is as high as 25%.

**Figure 4:**
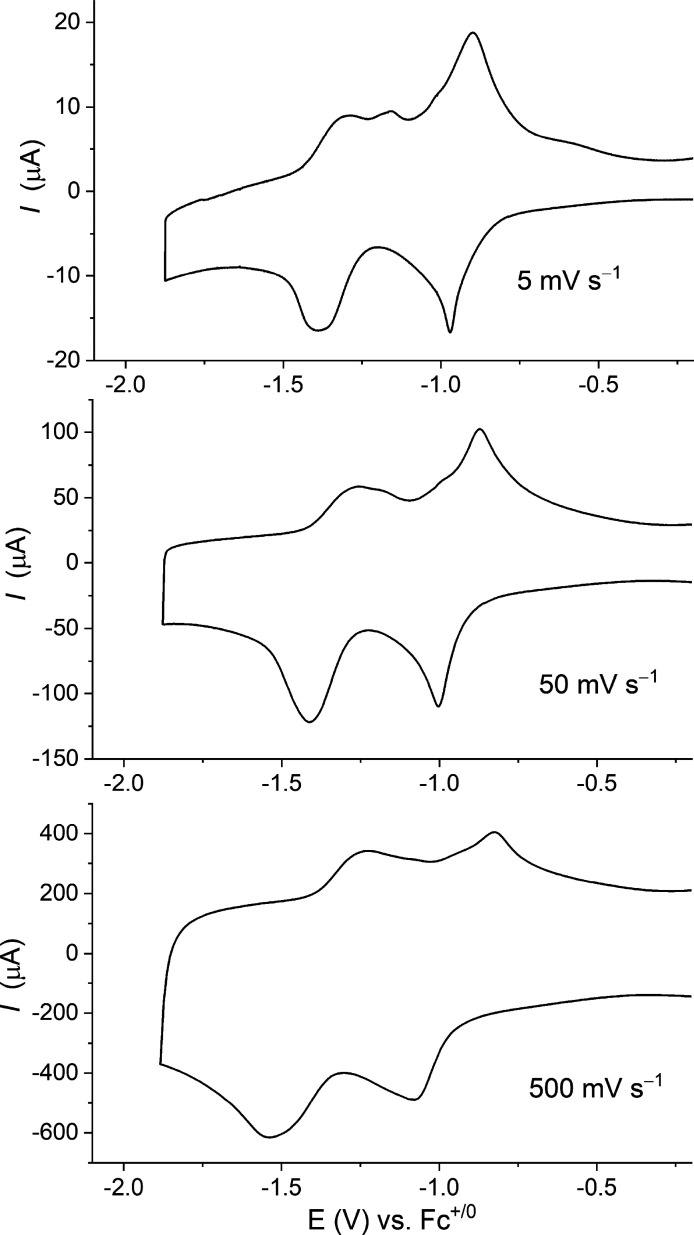
CVs of PCN-700_NDI_FeFe at different scan rates, showing dynamic changes of attractive interactions at high scan rates. Conditions: 0.5 M KPF_6_ in DMF.

The first reduction at *E*_1/2_ = −0.94 V corresponds to the first one-electron reduction of the NDI linker (NDI/NDI^•–^) in analogy to the assignment in PCN-700_NDI_ta. In the cathodic scan, this reduction is characterized by an unusual spike-like wave (width at half-height of 70.5 mV at ν = 50 mV s^–1^), a feature that has precedence in polymers with discrete redox-active sites that have been deposited as films on electrodes.^35^ The behavior is usually explained by the presence of attractive interactions within the material that disturb the Nernstian response that is governed solely by the ratio between the concentration of reduced and oxidized sites at the electrode surface. Furthermore, in the reports on polymer films, it was shown that reduction/oxidation of the redox-active sites induced dynamic changes in the film, which occur over the time scale of the CV.^36,37^ This results in phase-like behavior where microscopic domains form with differences in the concentration of solvent and ionic species. These effects will be more apparent if the scan rate is slow compared to this rate of interconversion, while, at faster scan rates, these processes would be outrun. Indeed, a similar effect was observed in the CVs of PCN-700_NDI_FeFe, where the first reductive wave transitions from a thin symmetric peak at low scan rates into a broad peak as the scan rate is increased (Figure 4). This behavior may result from dynamic changes in the solvation and ion content of the MOF pores associated with the change in redox state of the framework (reduction/oxidation of the redox-active linkers) augmenting the attractive interactions. Furthermore, the peak current of both reductive processes is proportional to ν^1/2^ at high scan rates, indicative of a semi-infinite diffusion response, while at lower scan rates finite diffusion is observed where the peak current transitions closer to a linear relationship with ν (see Figures S26, S27, and S28). The presence of these effects as known from redox polymers has not been described in MOFs before but is clearly observed in PCN-700_NDI_FeFe.

The second reductive feature in the CV of PCN-700_NDI_FeFe is broader than that in PCN-700_NDI_ta (best visible at higher scan rates in Figure 4), pointing to the presence of multiple redox processes at very similar potential. The Fe_2_ linker belongs to the bdt (benzene-1,2-dithiolato) series of [FeFe] model complexes and is known to exhibit inverted electrochemical reductions; i.e., the second reduction is more facile than the first one, ultimately leading to a single wave that corresponds to a two-electron reduction.^38^ This reduction is observed in the homogeneous linker at a similar potential (*E*_1/2_ = −1.23 V vs Fc^+/0^) as the NDI^•–^/NDI^2–^ couple (Figure 3b, Figure S24), and both processes can thus be expected to contribute to the second reduction wave in PCN-700_NDI_FeFe. However, the overall charge that is passed during the second reductive process does not correspond to three electrons, as one would expect for the two-electron reduction of the [FeFe] linker that is overlaid by the NDI^•–^/NDI^2–^ couple. Instead, integration of the current response at slower scan rates (5 mV s^–1^) reveals that a similar amount of charge is passed in both the first and second waves, observed at −0.94 and −1.34 V, respectively (see Figure S29).

This observation is important when investigating the role of the NDI as an electron mediator to the Fe_2_ site, as not all conceivable reductions may be visible in the CV of PCN-700_NDI_FeFe. With every linker reduction being associated with the uptake of a cation from the electrolyte (or expulsion of an anion from the MOF),^17,39^ there may be restrictions as to the total concentration of reduced linkers and cations that can be accommodated within the framework. Related effects have been observed by other groups. For example, Ameloot and Mayer reported that the degree of MOF reduction is governed by the presence of Na^+^ cations.^40^ On the anodic side, Farha and Hupp illustrated on Fc-NU-1000, a MOF consisting of pyrene-based linkers with incorporated ferrocene units, that the pyrene-based oxidation can be masked by preceding ferrocene oxidation. Only when the electrolyte concentration was increased, both oxidative processes become visible in the CV.^41^ Such effects can be rationalized based on microscopic electron transfer rates between linkers. In the simplest model, *D*_e_^app^ is proportional to the electron self-exchange rate between adjacent linkers^42,43^
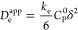
1where *C*_P_^0^ is the total concentration of redox-active linkers, δ is the average linker-to-linker distance over which electron transfer occurs, and *k*_e_ is the self-exchange rate constant. In the interior of the MOF pores, counterion association (pairing) may play an important role in stabilizing the products of these charge transfer reactions.^44^ If charge compensating ions are not present due to macroscopic transport limitations and/or size-exclusion effects, this would significantly raise the activation energy for electron transfer between linkers, resulting from a large local concentration of negative charges. Overall, such a situation would decrease the electron self-exchange rate and thus yield a low *D*_e_^app^ for a given redox couple, which then might have a vanishingly small current response.

In the case of PCN-700_NDI_FeFe, the hypothetical exhaustive four-electron reduction would correspond to an electron and cation concentration that is evidently beyond what the MOF can accommodate. In other words, as more and more charges are introduced into the MOF, electron and ion transport is slowed down, and as a result, the apparent diffusion coefficients *D*_e_^app^ that characterize these later reductions are greatly decreased. Interestingly, by increasing the scan rate for the CV of PCN-700_NDI_FeFe to a regime with a semi-infinite diffusion response (500 mV s^–1^), the charge that is passed during the second wave is increased by a factor of 2 relative to that of the first wave (Figure S30). This is consistent with an interpretation that the charging saturation that poses a threshold for the observed current density is less severe in the semi-infinite diffusion regime, i.e., when the CV is conducted at high scan rates. Availability of counterions may depend on macroscopic transport and will likely vary with scan rate. Given that the charge transport processes in the MOF are dynamic (electron/ion movement), it is likely that the self-exchange rate and therefore *D*_e_^app^ will also vary with the time scale of the electrochemical experiment, giving rise to nonlinear behavior. This phenomenon can be expected to be particularly severe in systems like PCN-700_NDI_FeFe, in which multiple reductions occur within a small potential window.

### Electrochemical Characterization in the Presence of Acid

In order to shine further light on the interplay between the NDI^•–^/NDI^2–^ and Fe_2_^0/2–^ couples, their voltammetric response in the presence of acid was probed. Acetic acid (AcOH) was chosen for this study because it is known to protonate the Fe_2_^2–^ state while not resulting in turnover which would complicate analysis.^38,45^ AcOH is also sufficiently small for MOF penetration, in contrast to, for example, tosylic acid which does not induce any changes in the MOF (see Figure S32).

**Figure 5:**
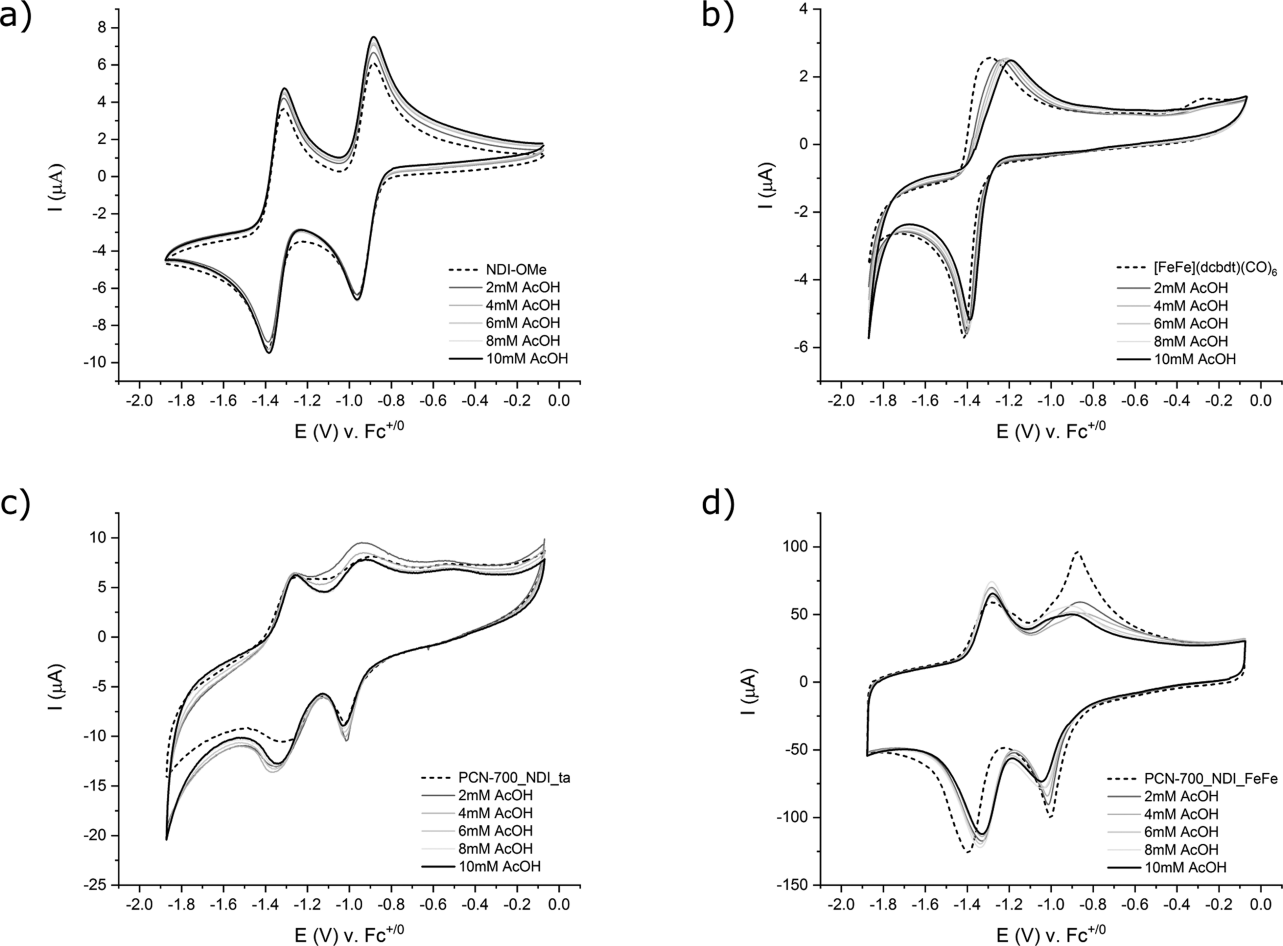
CVs of AcOH titrations of homogeneous functional linkers (a) NDI-OMe (1 mM), (b) [FeFe](dcbdt)(CO)_6_ (1 mM) and modified MOFs, (c) PCN_NDI_ta, and (d) PCN-700_NDI_FeFe. CV conditions: 0.5 M KPF_6_ in DMF. Scan rate: 50 mV s^–1^.

We first turned our attention to CVs of PCN-700_NDI_ta upon increasing concentrations of acetic acid (Figure 5c). As expected, the first reduction remains largely unaffected by addition of acid, and the peak potential remains unchanged. The peak potential of the second reduction wave experiences an unexpected shift of 45 mV to cathodic potentials, thereby rendering this reduction more similar to the peak potential of the homogeneous NDI linker (Figure 5a). The exact reason for this cathodic shift is unclear at present, but the important point for the subsequent discussion is that the peak does not shift *anodically*, which is the direction that is expected from protonation events that are coupled to prior reductions.

Upon addition of increasing amounts of AcOH to PCN-700_NDI_FeFe, various changes to the CV of the material can be observed (Figure 5d). First, the spike-like shape of the first reduction (NDI/NDI^•–^) changes to that of a normal diffusion-controlled process. Addition of acid thus appears to augment the interactions between reduced and oxidized NDIs that lead to the spike-like voltammetric response in the absence of acid. The peak potential of the second reductive process, which is assigned as the combined (NDI^•–^/NDI^2–^) and Fe_2_^0/2–^ couple, shifts as one wave to more positive potentials (Δ*E*_p,c_ = 70 mV). This response is expected for the Fe_2_^0/2–^ couple upon fast protonation and can be reproduced for the homogeneous linker (Δ*E*_p,c_ = 30 mV at 10 mM AcOH) (Figure 5b). The overall charge that is passed during the second reduction wave remains considerably less than the expected 3-electrons.

With the inverted formal potentials of the Fe_2_ unit, it is the second reduction of this linker that is the thermodynamic sink for all charge transfer processes at the applied potential. As the system operates in a regime with limited electron and cation uptake capacity, it is not surprising that the second NDI reduction is not observed as a separate event at more cathodic potential. Nevertheless, considering the closely spaced formal potentials of the NDI^•–^/NDI^2–^ and Fe_2_^0/2–^ couples, electron transport occurs most likely not only exclusively between NDI linkers and Fe_2_ sites but also between the two linkers.

While not being the main focus of this study, the catalytic activity of PCN-700_NDI_FeFe for the electrochemical generation of H_2_ was evaluated. Carbon mesh electrodes dropcasted with the same MOF/carbon black/Nafion ink as employed for the previous characterizations were subjected to controlled potential electrolysis for 2 h in a 0.1 M aqueous acetate buffer at pH 5. At an applied potential of −1 V (vs Ag/AgCl), visible bubble formation was observed at the electrode surface. Gas chromatographic analysis of the gas mixture in the headspace of the electrolysis cell identified H_2_ as the gaseous product, while no H_2_ could be detected with an identical electrode under identical conditions but in the absence of PCN-700_NDI_FeFe (see the Supporting Information for details, Figures S33–S35).

## Conclusions

In summary, we have prepared the first MOF that contains an enzyme active site model as well as an energy-matched redox mediator, thereby mimicking the situation in [FeFe] H_2_ases. NDI-to-FeFe distances in PCN-700_NDI_FeFe are in the same range as those between [4Fe4S] clusters in [FeFe] and [NiFe] H_2_ases and also between [4Fe4S] clusters and the redox-active Ni site in the latter H_2_ase (see Figure S8). In both cases, enzyme and MOF, it is the role of the electron mediator to transport electrons to an active site that is deeply buried within the protein or MOF matrix.

In [FeFe] H_2_ases, charge and mass transport are optimized for a single active site to operate at the highest possible rate. This sophistication is achieved by higher coordination sphere interactions between the [4Fe4S] clusters and an elaborate peptide environment. Consequently, enzymes are rather large constructs, with typical [FeFe] H_2_ases extending over many nanometers, approximately 8 × 6 × 5 nm^3^ for the [FeFe] H_2_ase from *Clostridium pasteurianum*.^1^ There is no biological necessity for electron transfer between [FeFe] H_2_ases.

This situation is vastly different in a MOF such as PCN-700_NDI_FeFe, in which catalytic sites are organized periodically throughout the framework. This arrangement allows for a high density of catalytic sites that, once prepared on conducting substrates, could give rise to industrially relevant current densities in electrocatalytic applications. The MOF design however comes at a cost, and charge transport is not similarly optimized as in the enzyme. In fact, overall catalytic efficacy is often limited by charge diffusion to supply all catalysts within a MOF with electrons to run at maximum turnover frequencies. In such cases, additional mediator linkers with a higher *D*_e_^app^ than the catalyst linker alone can remedy these shortcomings.

The present study indicates important features when analyzing MOFs with redox-active linkers by voltammetry. Attractive interactions have been identified in a MOF context for the first time, giving rise to unusually shaped CV waves. Also, apparent diffusion coefficients are not constants and may change depending on the relative concentrations of reduced/oxidized NDI linkers and charge-balancing counterions. This may be a result from restricted movement of charge-compensating redox-inactive counterions. Such nonlinear effects have hitherto been largely overlooked in MOFs for electrocatalytic applications but are predicted to be of crucial importance in the design of future (electro)catalysis systems.

## Supporting Information Available

Synthetic procedures, detailed characterization of linkers and MOFs, and cyclic voltammograms (PDF)

## Notes

The TOC figure includes a depiction of the crystal structure of [FeFe] hydrogenase (*Clostridium pasteurianum* CpI, PDB: 4XDC)^31^ that was created with NGL Viewer.^32^

## Abbreviations

ta: terephthalic acid
NDI: naphthalene diimide-*N*,*N*-bis(propanoate)
Me_2_tpdc: 2′,5′-dimethyl-[1,1′:4′,1″-terphenyl]-4,4″-dicarboxylate
Me_2_dpdc: 2,2′-dimethyldiphenyl 4,4′-dicarboxylate
dcbdt: 1,4-dicarboxylbenzene-2,3-dithiolate
AcOH: acetic acid
