# Supplementary material for: Mimicking the Electron Transport Chain and Active Site of [FeFe] Hydrogenases in One Metal–Organic Framework: Factors That Influence Charge Transport
Source: J Am Chem Soc. 2021 May 24;143(21):7991–9. doi: 10.1021/jacs.1c01361 (PMC8176456; doi:10.1021/jacs.1c01361)
Supplement: Supplementary file 1 — ja1c01361_si_001.pdf [file ja1c01361_si_001.pdf]

Supporting Information

Mimicking the Electron Transport Chain and Active Site of [FeFe] Hydrogenases in one Metal-Organic Framework: Factors that Influence Charge Transport

Ashleigh T. Castner,[a] Ben A. Johnson,[a] Seth M. Cohen,[b] Sascha Ott[a]

[a]Department of Chemistry - Ångström Laboratory, Uppsala University, Box 523, 75120 Uppsala, Sweden.
[b]Department of Chemistry and Biochemistry, University of California, La Jolla, San Diego, CA 92023-0358, USA.

Table of Contents

1) General Materials and Methods 2

2) Linker Synthesis and Characterization 3

- Linker synthesis procedures 3
- ^{1}H and ^{13}C NMRs for prepared linkers 6
- ATR-FTIR of linkers for SLI 11

3) MOF Preparation and Characterization 12

- Procedures for MOF prepartion and characterization 12
- Image of MOF materials and estimation of linker distances 13
- ^{1}H NMR of digested MOF materials 14
- BET analysis 17
- SEM images 20

4) MOF-modified Electrode Preparation and Electrochemical Characterization 21

- Preparation of MOF-modified electrodes 21
- CVs of homogeneous linkers 22
- Scan rate analysis of modified MOFs 24
- Double logarithm plots of modified MOFs 26
- Integration of current: PCN-700_NDI_FeFe CVs 27
- Electrochemical stability: PCN_NDI_FeFe 28
- Tosylic acid addition to PCN-700_NDI_FeFe 28
- Electrolysis and H_2 Detection 29

1) General Materials and Methods

All solvents and chemicals were purchased from commercial suppliers (Sigma Aldrich, VWR, Fluorochem, TCI) and were used without further purification.

^{1}H and ^{13}C NMR spectra were measured on a JEOL 400 MHz spectrometer at 293K. The chemical shifts are reported in ppm and are internally referenced to the residual solvent peak. ATR-FTIR data was obtained on a Bruker ALPHA FTIR spectrometer at room temperature from 4000 to 650 cm^{-1}. Powder X-ray diffraction (PXRD) patterns were obtained on a Simons D5000 diffractometer (Cu Kα, λ = 0.15418 nm) from 3°-55° at 45 kV and 40 mA with a step size of 0.02°. Bulk powder samples were dried under active vacuum at room temperature prior to measurement. Scanning electron microscopy (SEM) images were obtained on a Zeiss LEO 1530 Schottky FEG scanning electron microscope equipped with InLens and secondary electron (SE2) detectors at an acceleration voltage of 3 kV or 4 kV. MOF powders were attached via conductive carbon tape to sample holder disks and loosely attached particles were removed with a stream of air before imaging. BET surface area measurements were performed with a Micromeritics ASAP 2060. N_2 adsorptions isotherms were collected at 77K (liquid N_2 immersion). The bulk MOF samples were activated at 85°C under active vacuum (1 x 10^{-4} Pa) using a Micromeritics SmartVacPrep sample preparation unit prior to BET measurement. Metal content for PCN-700_NDI_FeFe MOF samples was determined by inductively coupled plasma optical emission spectroscopy (ICP-OES) using a Perkin Elmer Avio 200 ICP-OES setup with Syngistix software.

Cyclic voltammetry (CV) was performed using an Autolab PGSTAT100 potentiostat with GPES 4.9 software (EcoChemie). A 0.5 M solution of KPF_6 in DMF was used as the supporting electrolyte. A 3-electrode setup was employed with a glassy carbon rod counter electrode and a nonaqueous Ag/Ag(NO_3) (10 mM in MeCN) reference electrode, referenced to the ferrocenium/ferrocene couple ($Fc^{+/0}$). The counter and reference electrodes were isolated from the main solution by salt bridges with glass frit tips and filled with the supporting electrolyte solution. MOF-modified GC electrodes were employed as the working electrode for MOF samples. Solution-phase measurements of the linkers were performed at a 1 mM concentration in DMF with 0.5 M KPF_6 as supporting electrolyte using a GC working electrode (0.071 cm^2). Prior to measurements, the solutions were sparged with Ar to remove O_2. CVs were measured in 5 mL of supporting electrolyte solution at a scan rate of 50 mV/s. AcOH titration was performed by adding neat glacial acetic acid directly to the electrolyte solution and stirring.

2) Linker Synthesis and Characterization

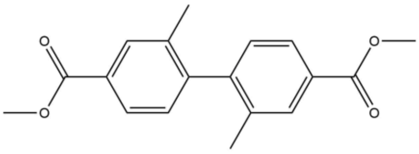

(1)

Synthesis of **(1)**. To 30 mL of DMF (degassed by 3 rounds of freeze-pump-thaw) was added methyl 4-iodo-3-methyl benzoate (1.380 g, 5.0 mmol), bis(pinacolato)diboron (1.395 g, 5.5 mmol), potassium acetate (1.470 g, 15 mmol), and [1,1'-bis(diphenylphosphino)ferrocene]dichloropalladium(II) (Pd(dppf)Cl_2) (0.150 g, 0.18 mmol). The reaction was stirred at 85°C overnight under Ar. The reaction was allowed to cool to RT, and methyl 4-iodo-3-methyl benzoate (2.07 g, 7.5 mmol), 2M (aq) Na_2CO_3 (12.5 mL) was added. The crude reaction mixture was degassed by freeze-pump-thaw (3x), and Pd(dppf)Cl_2 (0.150 g, 0.018 mmol) added to the solution. The reaction mixture was then stirred at 80°C overnight under Ar. The reaction was cooled to RT, and the solvents removed under vacuum. The resulting solid was diluted with brine (50 mL) and extracted with $CHCl_3$ (3 x 50 mL). The organic layers were collected and washed again with fresh brine. The organic layers were then collected, dried over Mg_2SO_4, filtered, and concentrated. The crude product was purified by flash chromatography on silica using a solvent gradient of 5-10% EtOAc in pentane. The final product was obtained as an off-white crystalline solid (1.175 g, 78.4%). ^1H NMR (400 MHz, $CDCl_3$) δ ppm: 7.97 (s, 2H), 7.91 (d, 2H), 7.16 (d, 2H), 3.95 (s, 6H), 2.08 (s, 6H). TOF-MS (+) (CH_2Cl_2/MeOH): $[M + H]^+$, m/z = 299.1348 (cald m/z = 299.3403); $[M + Li]^+$, m/z = 305.1565 (cald m/z = 305.2734)

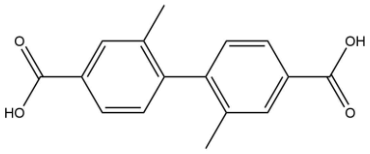

(2)

Synthesis of Me_2dpdc (dpdc = diphenyl dicarboxylate) **(2)**. **(1)** (1.1752 g, 3.94 mmol) was suspended in THF/MeOH (1:1, 30 mL) and 2M (aq) KOH (15 mL), and the reaction was stirred at 80°C overnight. The reaction was then cooled to RT, and the organics removed by rotary evaporation. The aqueous phase was then acidified with 1M (aq) HCl to produce a white precipitate. The precipitate was then filtered, washed with H_2O, and dried under vacuum to afford the pure product as a white solid (1.052 g, 98.8%). ^1H NMR (400 MHz, DMSO-d_6) δ ppm: 12.96 (s), 7.91 (s, 2H), 7.83 (d, 2H), 7.21 (d, 2H), 2.04 (s, 6H). ESI-MS (-) (MeCN/H_2O): $[M - H]^-$, m/z = 269.08122 (cald m/z = 269.2715)

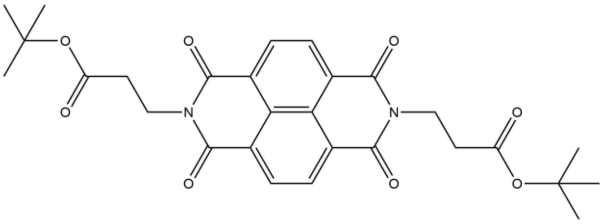

(3)

Synthesis of NDI-OtBu **(3)**. To a flame-dried, Ar-purged 2-neck RBF was charged 1,4,5,8-naphthalenetetracarboxylic dianhydride (0.6710 g, 2.5 mmol), and β-alanine *tert*-butyl ester hydrochloride (1.000 g, 5.5 mmol). The reactants were suspended in dry toluene (110 mL), and DIPEA

(1.1 mL, 6.255 mmol) was added. The vessel was fitted with a Dean-Stark trap, and refluxed overnight under Ar. The reaction was cooled to RT, and concentrated by rotary evaporation. The crude material was purified by flash chromatography on silica using a solvent gradient of 0-1% MeOH in DCM. The pure product was obtained as a pale yellow solid. (1.215 g, 92.8%). ^{1}H NMR (400 MHz, $CDCl_3$) δ ppm: 8.77 (s, 4H), 4.48 (t, 4H), 2.70 (t, 4H), 1.42 (s, 18H). TOF-MS (+) (CH_2Cl_2/MeOH): $[M + H]^+$, *m/z* = 523.2166 (cald *m/z* = 523.5531); $[M + Na]^+$, *m/z* = 545.1909 (cald *m/z* = 545.5349)

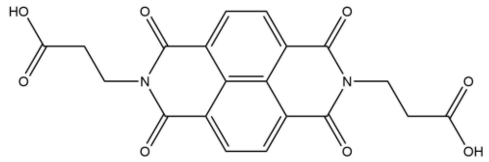

(4)

Synthesis of NDI-COOH **(4)**. **(3)** (1.215 g, 2.325 mmol) was added to a 2-neck 250 mL RBF and suspended in dry DCM (45 mL). TFA (5 mL) was added dropwise, and the reaction allowed to stir at RT overnight. The solvent was then removed under vacuum. The resulting solid was suspended in cold H_2O and filtered. The solid was further washed with cold H_2O and then dried to yield the pure product as a slightly yellow solid. (0.884 g, 88.5%). ^{1}H NMR (400 MHz, DMSO-d_6) δ ppm: 12.43 (s), 8.66 (s, 4H), 4.27 (t, 4H), 2.64 (t, 4H). TOF-MS (-) (CH_2Cl_2/MeOH): $[M - H]^-$, *m/z* = 409.0695 (cald *m/z* = 409.3253)

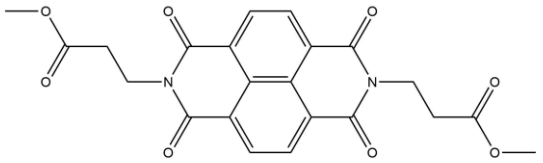

(5)

Synthesis of NDI-OMe **(5)**. To a flame-dried, N_2-purged RBF was charged 1,4,5,8-naphthalenetetracarboxylic dianhydride (0.5000 g, 1.86 mmol), and β-alanine methyl ester hydrochloride (0.5725 g, 4.10 mmol). The reactants were suspended in dry toluene (110 mL), and DIPEA (0.812 mL, 4.66 mmol) was added. The vessel was fitted with a Dean-Stark trap, and refluxed overnight under N_2. The reaction was cooled to RT, and concentrated by rotary evaporation. The crude material was purified by flash chromatography on silica using a solvent gradient of 0-5% MeOH in DCM. The pure product was obtained as a pale yellow solid. (0.7370 g, 90.2%). %). ^{1}H NMR (400 MHz, $CDCl_3$) δ ppm: 8.77 (s, 4H), 4.53 (t, 4H), 3.70 (s, 6H), 2.80 (t, 4H). TOF-MS (+) (CH_2Cl_2/MeOH): $[M + H]^+$, *m/z* = 439.1146 (cald *m/z* = 439.3941)

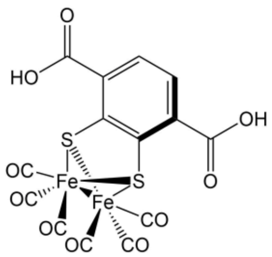

(6)

<u>Synthesis of [FeFe](dcbdt)(CO)$_6$ (dcbdt = 1,4-dicarboxylbenzene-2,3-dithiolate).</u> The preparation was slightly modified from a previously described method.[1] TMEDA (4.2 mL, 28 mmol) was charged into a flame-dried, Ar-purged 250 mL RBF and cooled to 0°C. *n*-BuLi (17.5 mL (1.6M in hexanes), 28 mmol) was added dropwise and stirred for ~10 minutes. 1,2-benzenedithiol (0.41mL, 3.56 mmol), dissolved in 30 mL dry cyclohexane, was added dropwise. The reaction was stirred at 0°C for 1.5 hours before allowing to slowly warm up to RT. The reaction was further stirred at RT under Ar for ~4.5 days. The reaction was then cooled to 0°C, and CO_2 (dried through P_2O_5) was bubbled into the reaction mixture for 3 hours. The crude reaction mixture was then concentrated and the resulting solids redissolved in dry THF (~50 mL). Triiron dodecacarbonyl (3.5 g, 6.95 mmol) was added and the reaction mixture set to reflux under Ar for ~1 hour. The reaction mixture was then concentrated with silica gel and the product purified by flash chromatography on silica with a 3 solvent gradient. The column is first run in pure pentane to isolate the bdt complex. The (mcbdt) complex is then eluted from the column with pure EtOAc. Finally, the desired (dcbdt) complex is eluted with 80:20 EtOAc:MeOH, and finally the column is flushed with 50:50 EtOAc:MeOH to ensure elution of all complex on the column. The appropriate fractions are collected and concentrated to yield the (dcbdt) complex as a dark red solid.

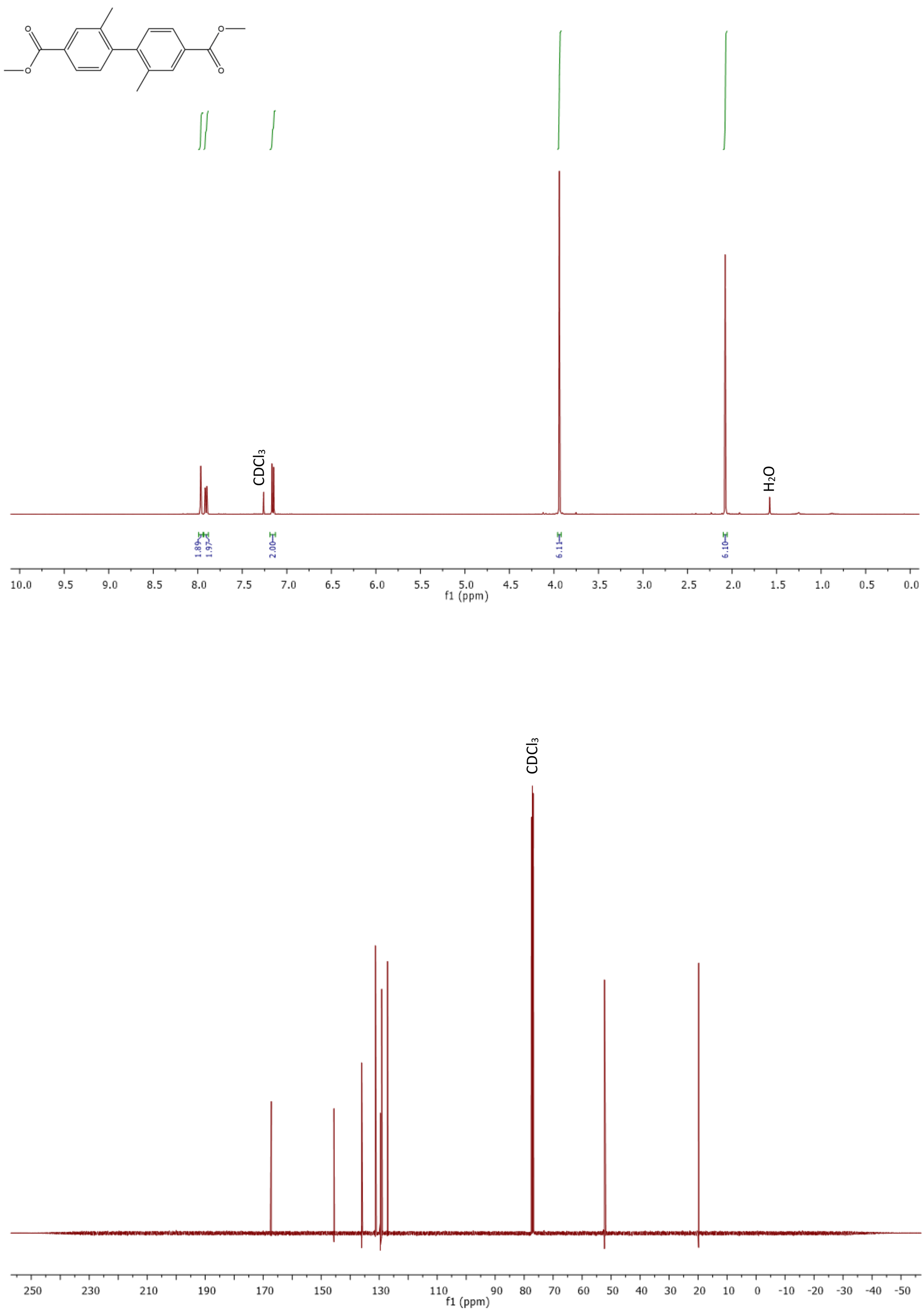

Figure S1. ^{1}H and ^{13}C NMR spectra of **1**.

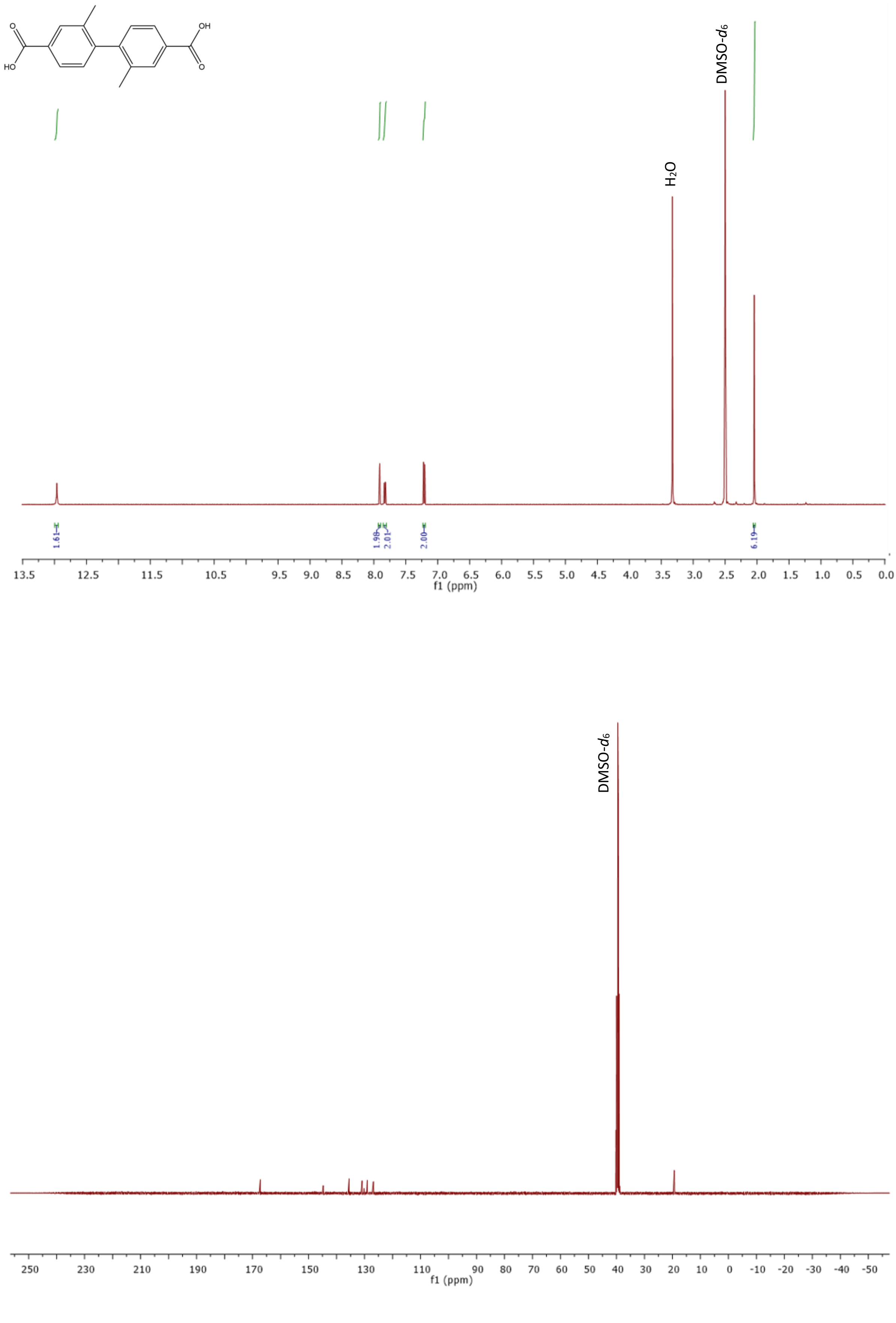

Figure S2. ^1H and ^{13}C NMR spectra of **2** (Me_2dpdc).

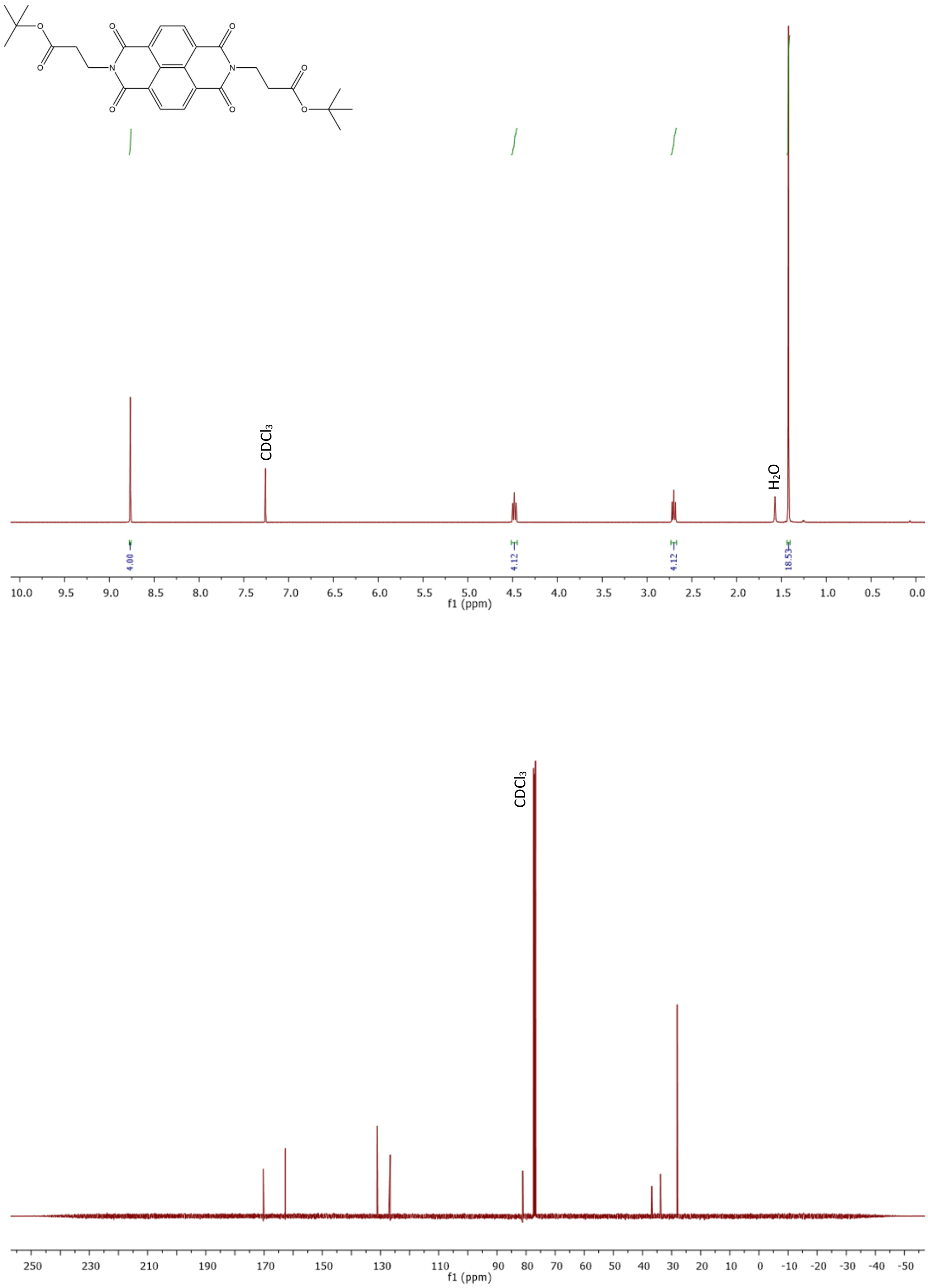

Figure S3. ^{1}H and ^{13}C NMR spectra of **3** (NDI-OtBu).

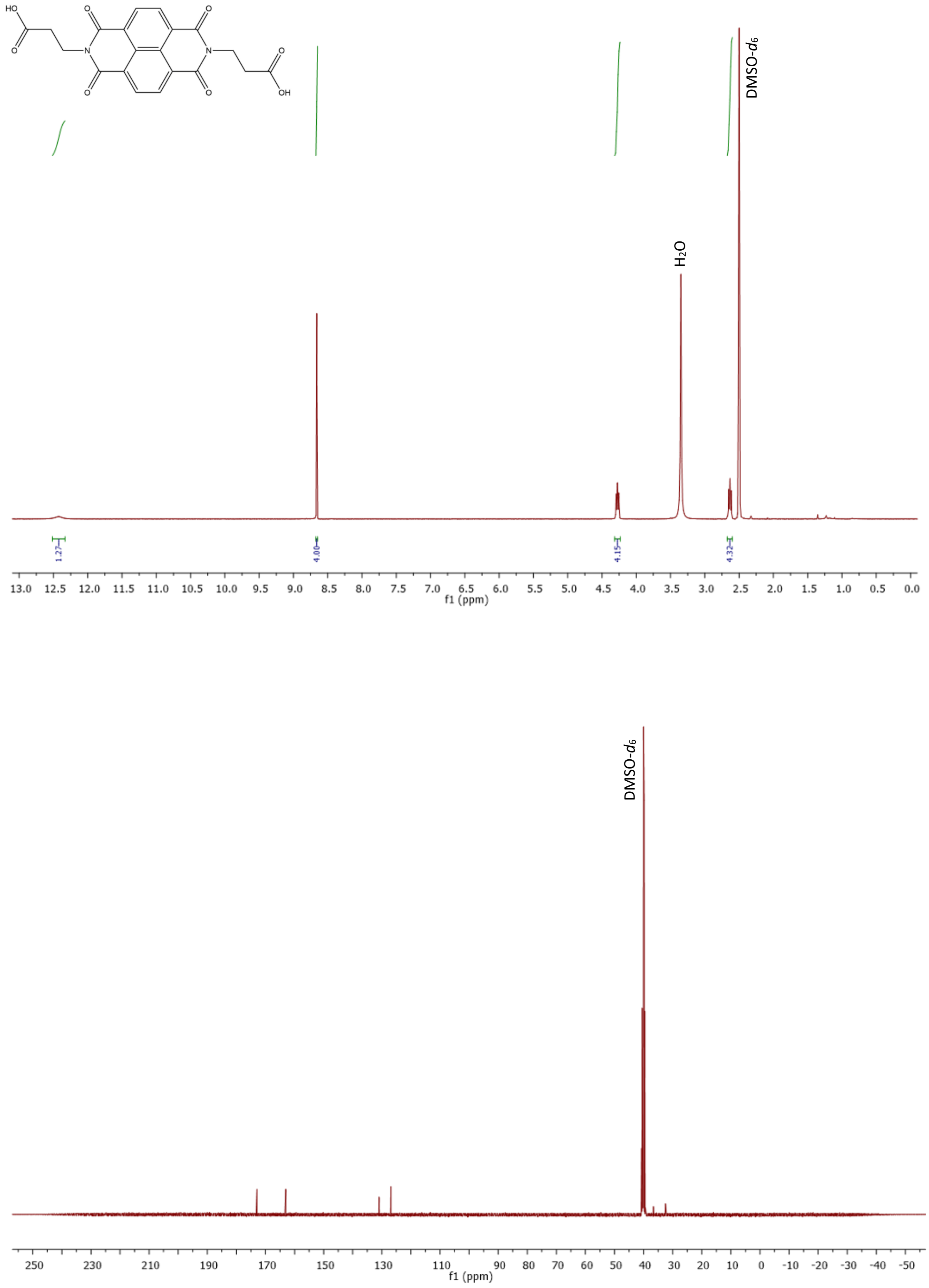

Figure S4. ^{1}H and ^{13}C NMR spectra of **4** (NDI-COOH).

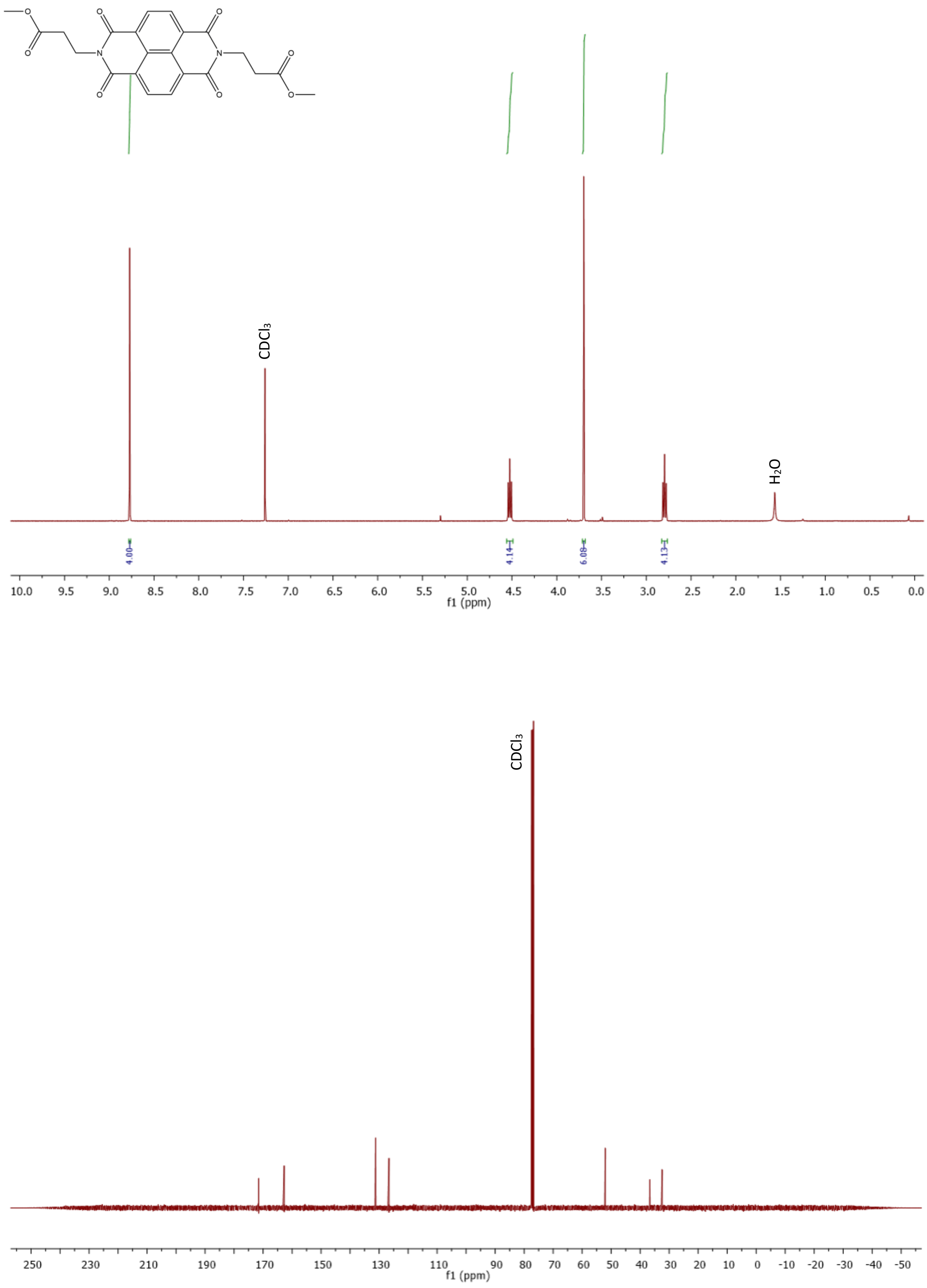

Figure S5. ^{1}H and ^{13}C NMR spectra of **5** (NDI-OMe).

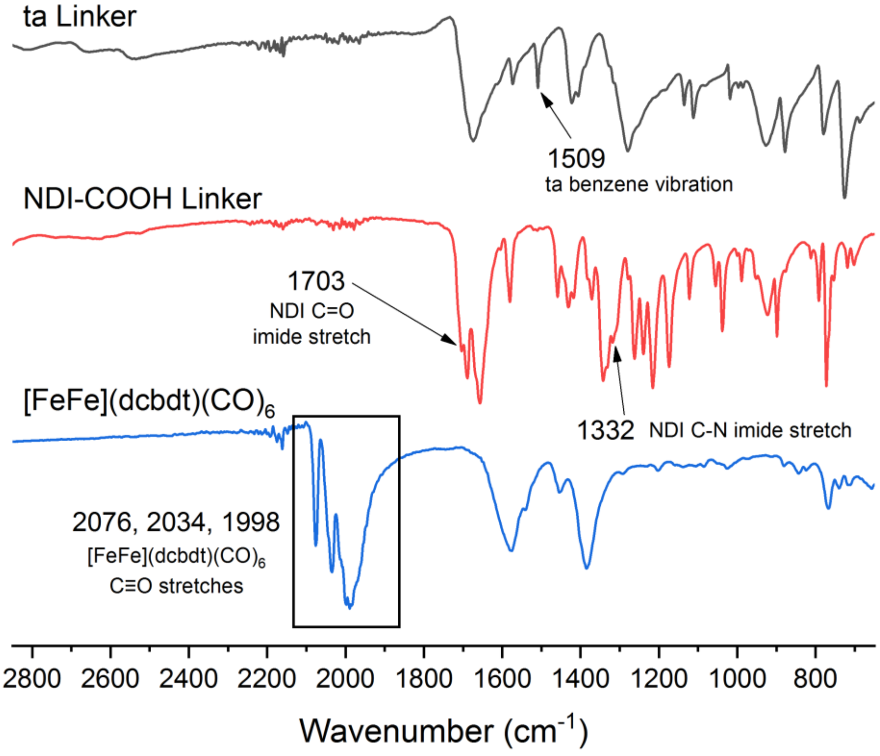

Figure S6. ATR-FTIR of unincorporated linkers (ta, NDI-COOH, and [FeFe](dcbdt)$(CO)_6$) with identifying IR signatures assigned for verification of linker incorporation in PCN-700.

3) MOF Preparation and Characterization

PCN-700 MOF Preparation. To Me_2dpdc linker **(2)** (0.180 g, 0.67 mmol) and $ZrCl_4$ (0.120 g, 0.51 mmol) was added DMF (11.45 mL) and TFA (0.55 mL, 7.18 mmol). The mixture was sonicated to homogeneously suspend the solids and 2 mL aliquots of this suspension were charged into 4 mL vials. Solvothermal MOF synthesis was initiated by incubation of these aliquots at 120°C for 72 hours. The resulting MOF crystals were washed with fresh DMF (3x) and stored in DMF until ready for use.

SLI 1: NDI-COOH into PCN-700 **(PCN-700_NDI)**. PCN-700 (dried under vacuum) and NDI-COOH (10 eq) were charged into a 20 mL vial and suspended in 8 mL DMF by gentle aspiration with a pipette. The resulting suspension was incubated at 75°C for 24 hours. After incubation, the MOF was soxhlet extracted with acetone for at least 18 hours to wash out any unincorporated NDI-COOH.

SLI 2.1: ta into PCN-700_NDI **(PCN-700_NDI_ta)**. PCN-700_NDI (dried under vacuum) and ta (4 eq) were charged into a 20 mL vial and suspended in 8 mL DMF by gentle aspiration with a pipette. The resulting suspension was stirred at RT for 24 hours. After linker incorporation, the MOF was soxhlet extracted with acetone for at least 18 hours to wash out any unincorporated ta or NDI-COOH.

SLI 2.2: [FeFe](dcbdt)$(CO)_6$ into PCN_NDI **(PCN-700_NDI_FeFe)**. PCN-700_NDI (dried under vacuum) and [FeFe](dcbdt)$(CO)_6$ (4 eq) were charged into a 20 mL vial and suspended in 8 mL degassed H_2O. The resulting suspension was stirred at RT for 24 hours in the dark and under Ar. After incorporation, the MOF was washed by suspending in fresh degassed H_2O for at least 3 hours in the dark (3x). The MOF was then washed with degassed acetone by suspending in fresh solvent in the dark, and the solvent exchanged until no color observed in the wash solvent.

^{1}H NMR of digested MOF samples. Prior to ^{1}H NMR analysis, MOF (~5 mg) was charged into a 4 mL vial with DMSO-d_6 (0.55 mL) and 35% DCl in D_2O (0.05 mL). The MOF suspension was sonicated for at least 20 minutes or until all MOF material was digested. The resulting solution was transferred directly to an NMR tube for analysis.

Digestion of PCN-700_NDI_FeFe for ICP-OES. Prior to analysis, MOF (~5 mg) was charged into a 2 mL microwave vial with 4:1 solution of 69% HNO_3 : 30% H_2O_2 (1 mL total volume). The vial was sealed and the MOF was digested in a microwave reactor for 10 minutes at 160°C. The solution was allowed to cool to room temperature, then was diluted to a total volume of 5 mL with H_2O (HPLC-LC-MS grade). This solution was then passed through a 0.2 µm syringe filter and used for ICP-OES analysis.

Estimation of NDI-to-FeFe distance in PCN-700_NDI_FeFe. From the crystal structure of PCN-703 (CCDC deposition number: 1036877)[2] obtained from the Cambridge Crystallographic Data Centre, the representative MOF structure with Me_2tpdc and ta linkers was viewed in the VESTA software. Estimated distances were obtained by measuring the distance between the 4' C of the Me_2tpdc linker (approximately where the imide N would be) to the nearest H's of the ta linker. This position would correspond to where the S atoms of the Fe_2 complex would be, but is the closest reference point to measure in the crystal structure. The estimated distances here are similar to the distances for electron transport in [FeFe] H_2ases and [NiFe] H_2ases between the [4Fe4S] clusters from 11-13Å.[3] Additionally, a recent report employing the PCN-700 MOF as a scaffold to construct a fluorescence sensor cites a distance of 14Å between incorporated fluorophore and recognition moieties,[4] which is also in the same distance regime estimated for PCN-700_NDI_FeFe from the PCN-703 structure.

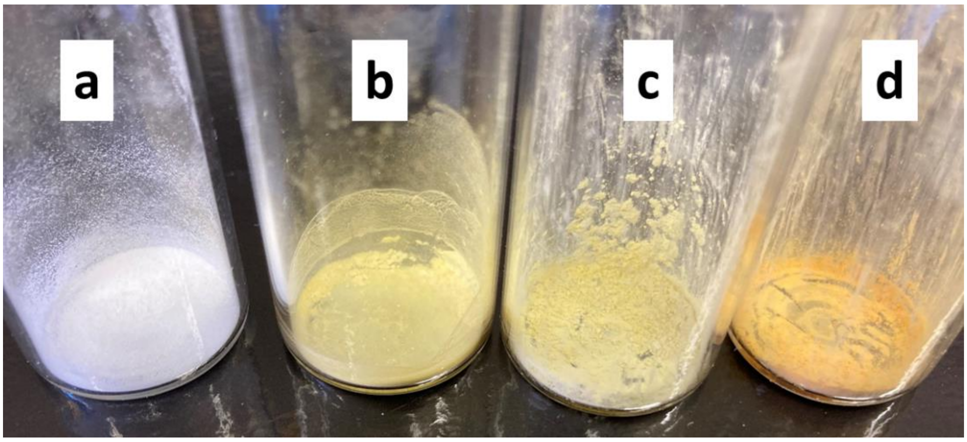

Figure S7. Photograph of MOF materials at each stage of SLI treatments. Pristine PCN-700 (a) is white in color. A color change is observed for PCN-700_NDI (b) to yellow following NDI incorporation and washing via soxhlet extraction with acetone. PCN-700_NDI_ta (c) remains yellow after sequential ta incorporation and washing. Incorporation of [FeFe](dcbdt)$(CO)_6$ into PCN-700_NDI results in an additional color change to orange for the MOF PCN-700_NDI_FeFe (d) after linker insertion and washing.

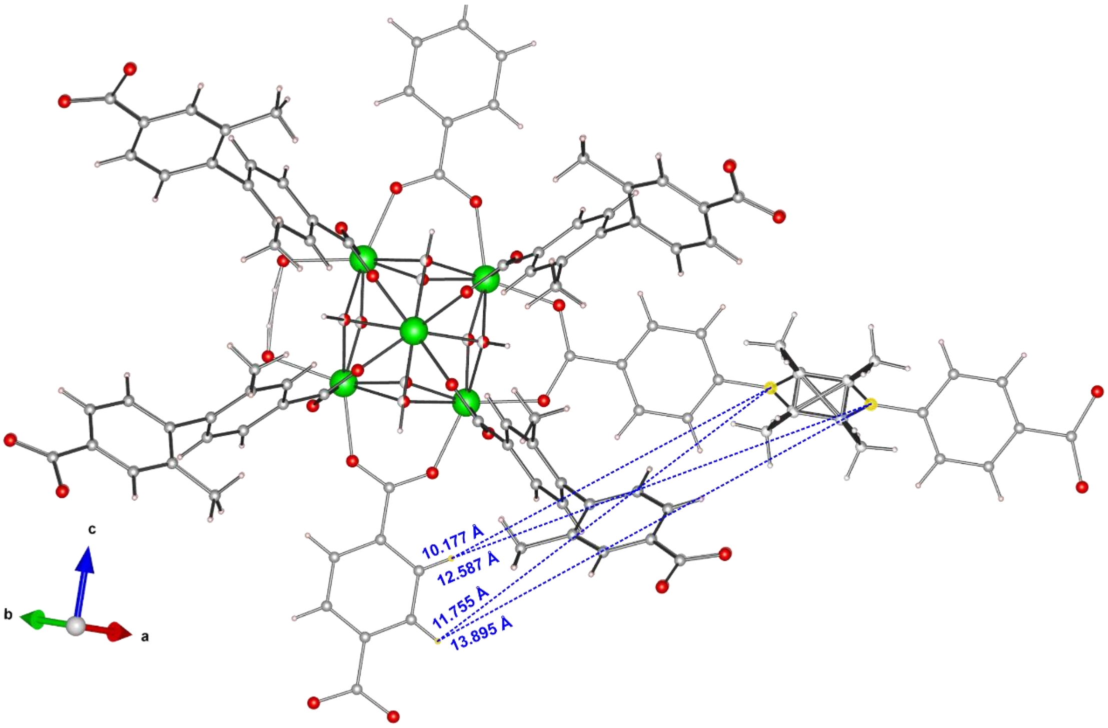

Figure S8. Crystal structure of PCN-703 (CCDC deposition number: 1036877),[2] corresponding to PCN-700 with Me_2tpdc and ta linkers incorporated. Distances measured would approximate distance for electron transfer in MOF PCN-700_NDI_FeFe.

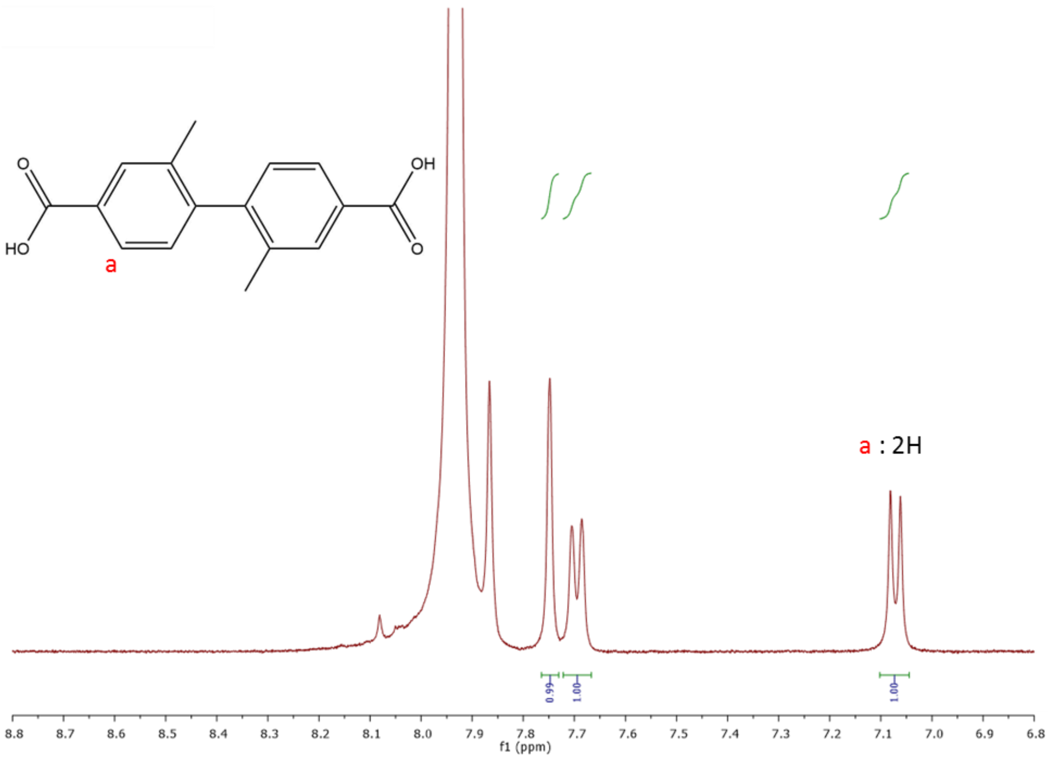

Figure S9. ^{1}H NMR spectrum of PCN-700 (5 mg MOF digested in 50 µL 35% DCl in D_2O / 550 µL DMSO-d_6).

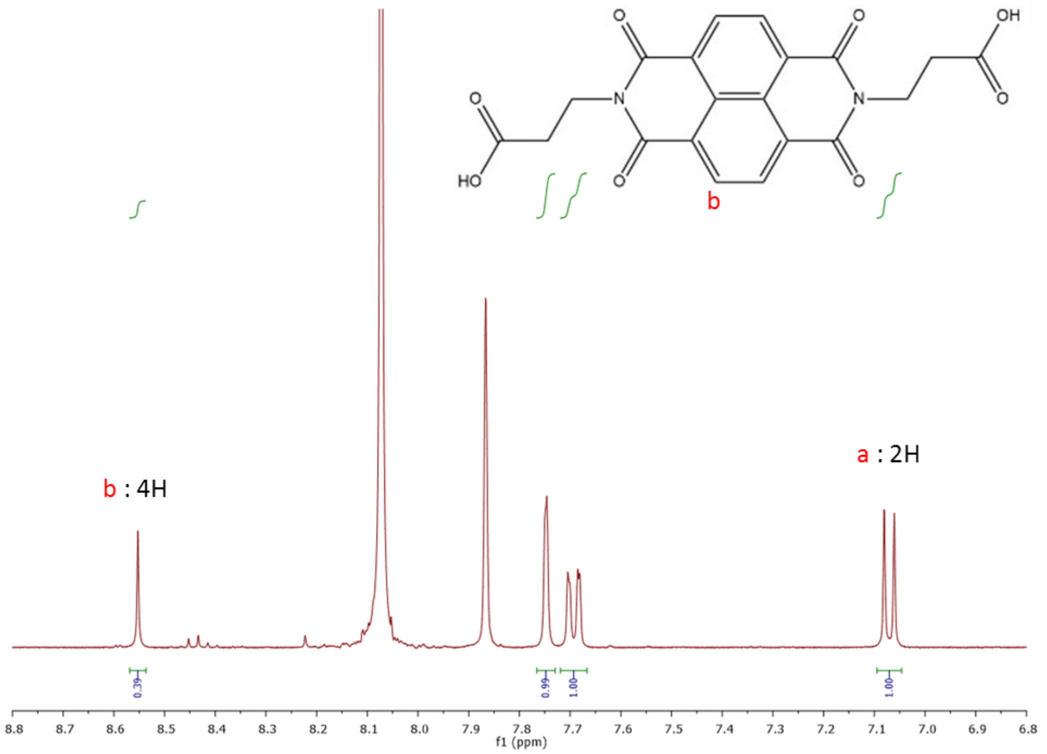

Figure S10. ^{1}H NMR spectrum of PCN-700_NDI (5 mg MOF digested in 50 µL 35% DCl in D_2O / 550 µL DMSO-d_6).

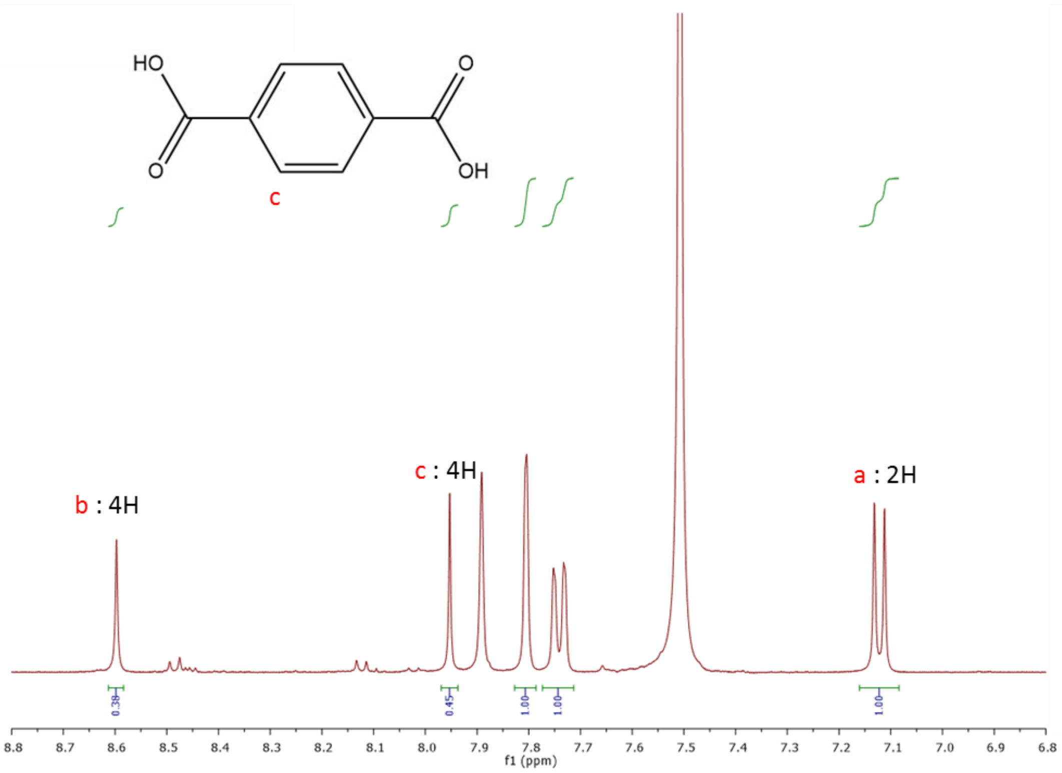

Figure S11. ^{1}H NMR spectrum of PCN-700_NDI_TA (5 mg MOF digested in 50 µL 35% DCl in D_2O / 550 µL DMSO-d_6).

MOF	Ideal linker ratio expected from single crystal structure	Linker ratio determined from ^{1}H NMR of digested MOF sample
PCN-700	All linkers Me_2dpdc	100% Me_2dpdc<br>**(Figure S9)**
PCN-700_NDI	Me_2dpdc : NDI-COOH → 8 : 2	Me_2dpdc : NDI-COOH = $\frac{1.00}{2H}$: $\frac{0.39}{4H}$ = **8 : 1.56**<br>(78% NDI incorporation)<br>**(Figure S10)**
PCN-700_NDI_ta	Me_2dpdc : NDI-COOH : ta → 8 : 2 : 2	Me_2dpdc : NDI-COOH : ta = $\frac{1.00}{2H}$: $\frac{0.38}{4H}$: $\frac{0.45}{4H}$ = **8 : 1.52 : 1.8**<br>(76% NDI incorporation, 90% ta incorporation)<br>**(Figure S11)**

Table S1. Percent linker incorporation in PCN-700 during SLI procedure.

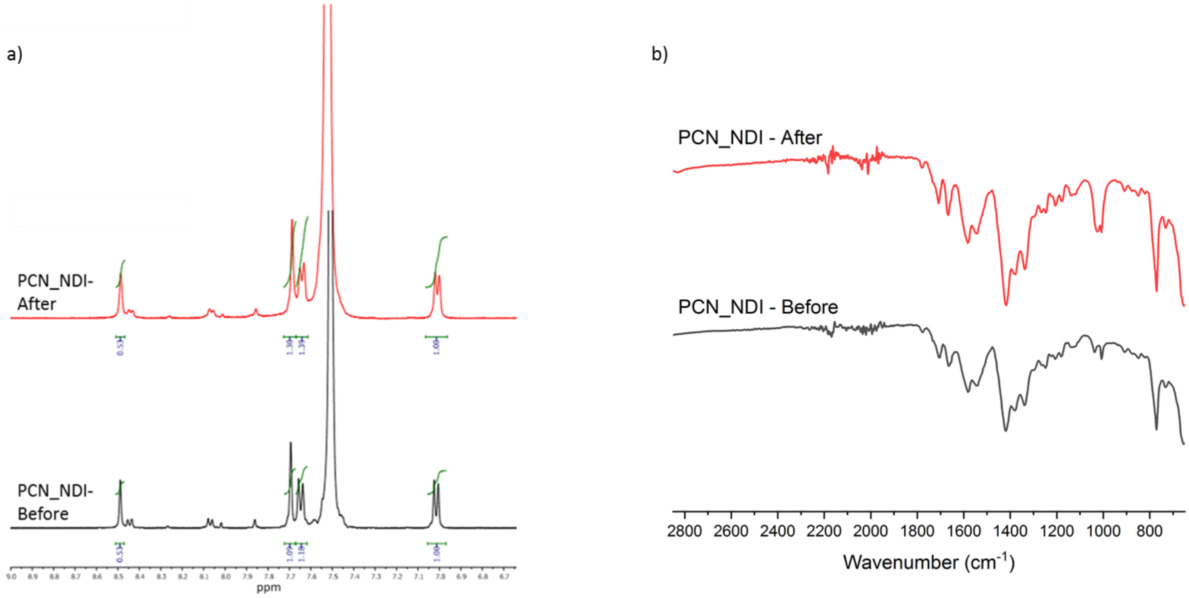

Figure S12. Control SLI of [FeFe](bdt)$(CO)_6$ in PCN_NDI. In the absence of coordinating carboxylates, the [FeFe](bdt)$(CO)_6$ complex cannot be incorporated under almost the same conditions as those used for SLI of the dicarboxylated complex [FeFe](dcbdt)$(CO)_6$ (deoxygenated MeOH instead of H_2O for complex solubility). [FeFe](bdt)$(CO)_6$ is removed from PCN_NDI during the typical washing steps to remove complexes trapped in the pores. This is demonstrated by comparing both (a) ^{1}H NMR of the digested MOF and (b) ATR-FTIR before and after treatment of PCN_NDI with the control complex [FeFe](bdt)$(CO)_6$. The absence of singlets at 7.2 and 6.8 ppm (characteristic of the [FeFe](bdt)$(CO)_6$ complex) in the ^{1}H NMR spectra after exposure of PCN_NDI to the control complex [FeFe](bdt)$(CO)_6$ ((a), red spectrum) confirm the absence of the bdt bridge after washing. The absence IR peaks at ~2076, 2034, 1998 cm^{-1} after complex exposure ((b), red spectrum) further demonstrates the absence of the [FeFe](bdt)$(CO)_6$ complex.

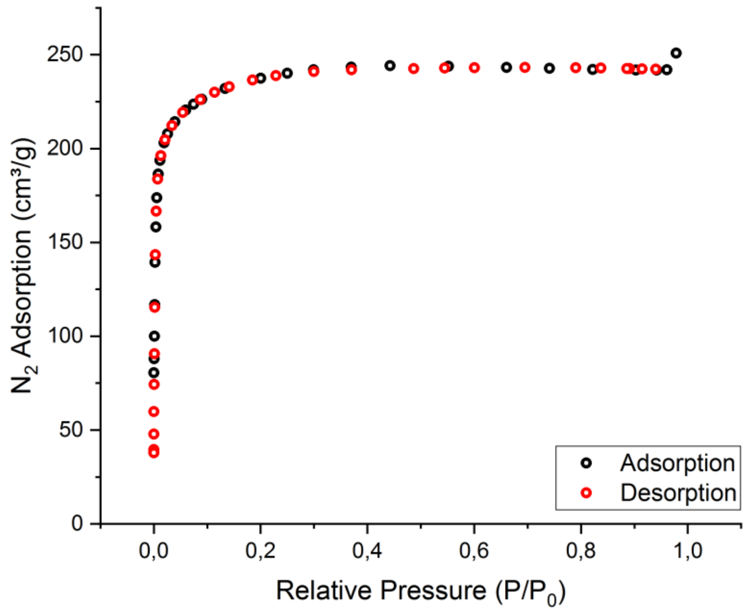

Figure S13. N_2 adsorption isotherm for PCN-700 (77K). Type I isotherm type obtained as is expected for PCN-700.

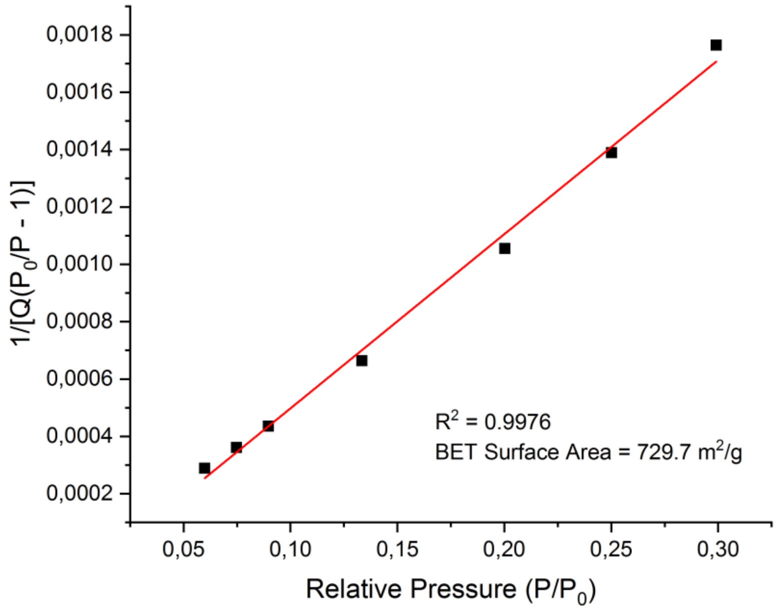

Figure S14. Linear BET plot from N_2 adsorption isotherm of PCN-700.

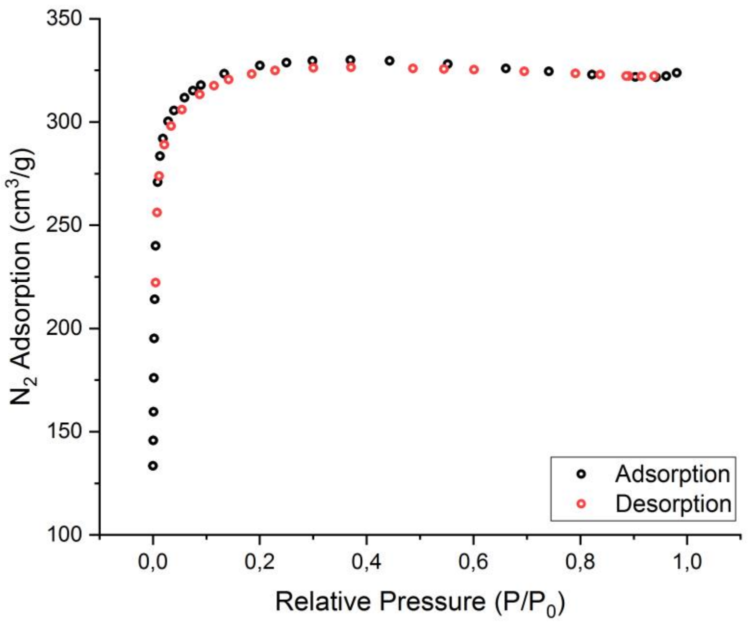

Figure S15. N_2 adsorption isotherm for PCN-700_NDI_ta (77K). Type I isotherm type obtained as is expected for dual linker incorporation into PCN-700.

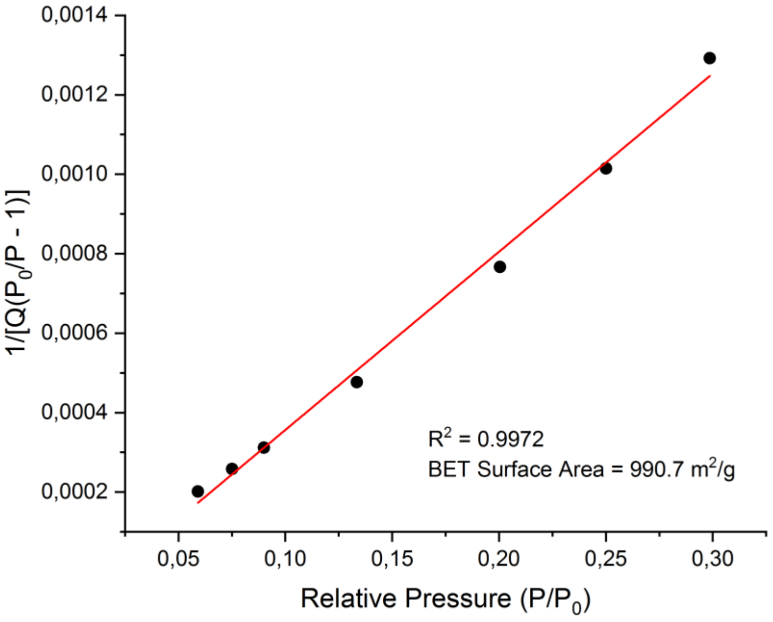

Figure S16. Linear BET plot from N_2 adsorption isotherm of PCN-700_NDI_ta.

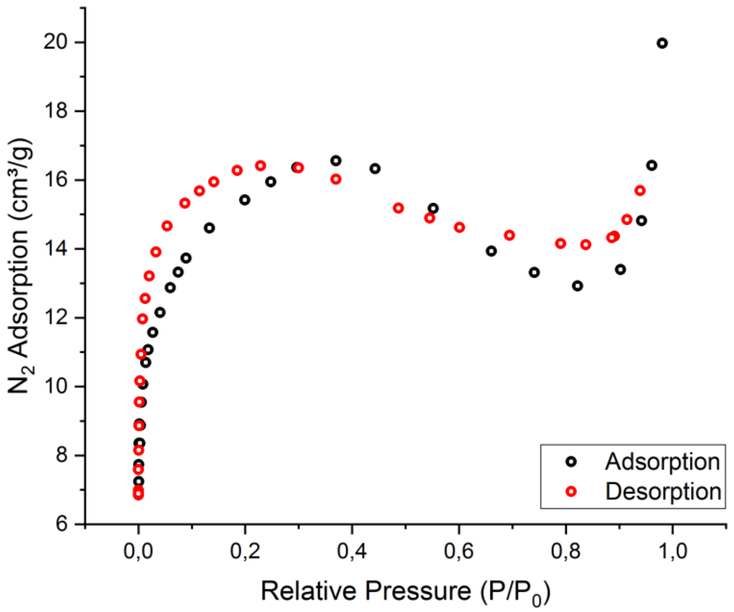

Figure S17. N_2 adsorption isotherm for PCN-700_NDI_FeFe (77K). Type IV isotherm type with hysteresis obtained. This is indicative of capillary condensation of the adsorbent N_2 molecules.[5] As the incorporated Fe_2 complex is not thermally stable, it is possible that complex degradation as a result of MOF activation at 85°C may result in pore clogging leading to this isotherm type.

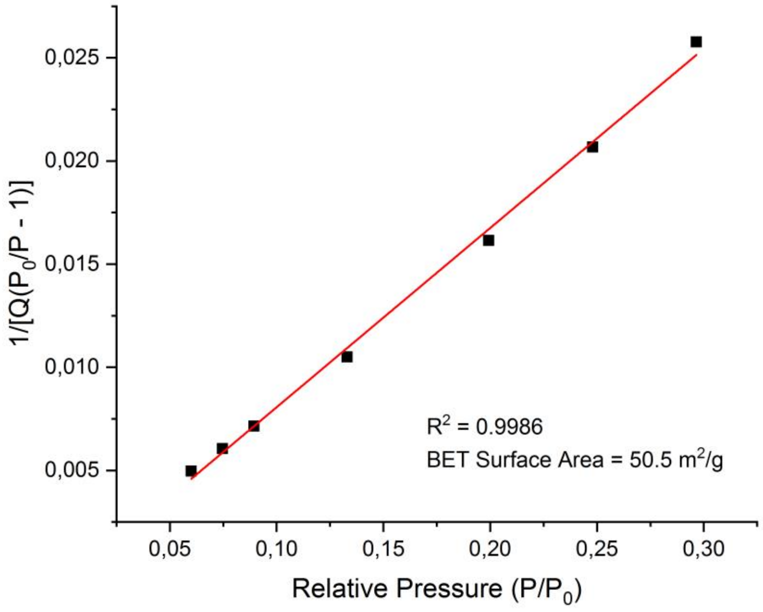

Figure S18. Linear BET plot from N_2 adsorption isotherm of PCN-700_NDI_FeFe.

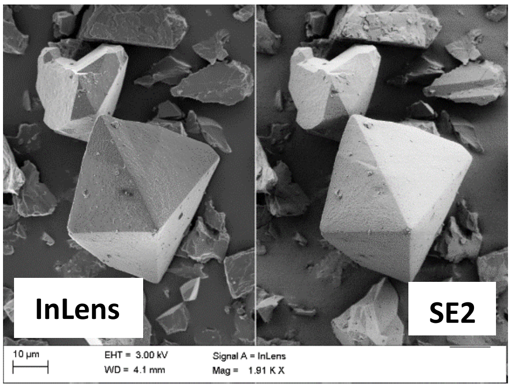

Figure S19. Example SEM image of PCN-700 MOF.

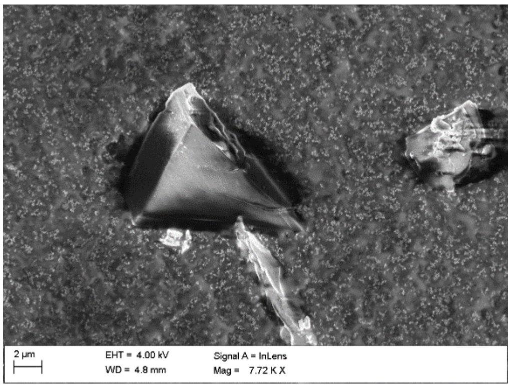

Figure S20. Example SEM image of PCN-700_NDI_ta MOF.

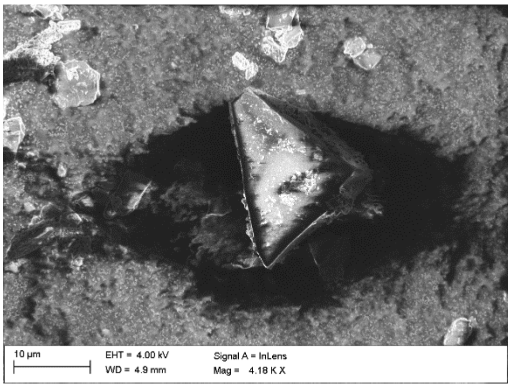

Figure S21. Example SEM image of PCN-700_NDI_FeFe MOF.

4) MOF-modified Electrode Preparation and Electrochemical Characterization

MOF-modified Electrode Preparation. MOF-modified electrodes were prepared by dropcasting similar to a previously described method.[6] Glassy carbon discs (7mm diameter) were used as working electrodes. Prior to MOF deposition, GC electrodes were carefully polished with sequentially smaller alumina particles down to 0.05 µm, rinsed thoroughly, sonicated in EtOH for 3 minutes, and dried. Electrodes were wrapped with Teflon tape to leave only the working area exposed prior to MOF deposition. A suspension of MOF (either PCN-700_NDI_ta or PCN-700_NDI_FeFe) was prepared for dropcasting onto GC working electrodes as follows: the desired MOF (1 mg) and carbon black (4 mg) was suspended in an aliquot of 2-propanol (0.5 mL) containing 0.5% v/v of Nafion® 117. The suspension was then homogenized by sonication for at least 20 minutes to form a stable MOF 'ink'. The ink was then dropcast (10 µL) onto polished GC electrodes and dried in air overnight.

Electrolysis for HER. Carbon mesh paper (cut to ~1x2 cm size) was used as the working electrode for MOF 'ink' deposition. The same procedure for MOF 'ink' preparation with PCN_NDI_FeFe described above was used, and 40 µL of this 'ink' was painted onto ~1 cm^2 of the carbon mesh electrode. The background 'ink' was composed of only carbon black and nafion. Following 'ink' deposition, the electrodes were dried overnight in air. For electrolysis in aqueous conditions at pH 5, a 0.1 M acetate buffer containing 0.5 M KCl was used as the supporting electrolyte. Electrolysis was performed in a 2-compartment H cell (Pine Research) separated by a glass frit. A Pt coil was used as the counter electrode, and an aqueous Ag/AgCl (4M KCl) electrode was used as the reference electrode. Both compartments were purged with Ar for at least 30 minutes before electrolysis. A potential of -1 V v. Ag/AgCl was then applied to the sparged and sealed system for 2 hours. After 2 hours, 80 µL of the headspace gas in the working compartment was sampled with a gas-tight syringe and injected into a GC for H_2 detection. The volume of the headspace in the working compartment was determined after electrolysis was complete.

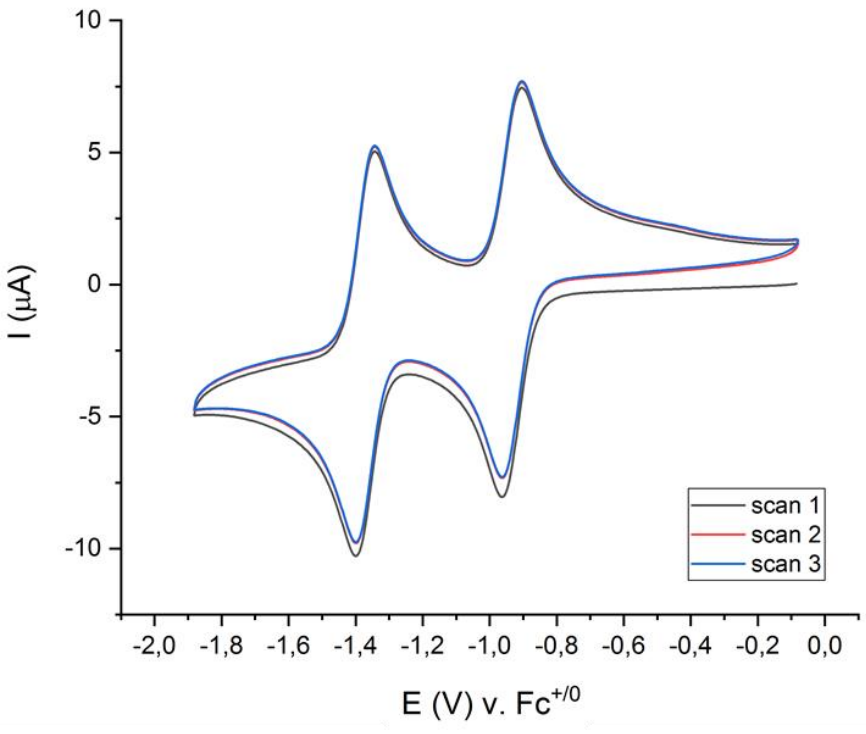

Figure S22. CV of homogeneous NDI-OtBu (1mM). Supporting electrolyte: 0.5M KPF_6 in DMF. Scan rate: 50 mV s^{-1}.

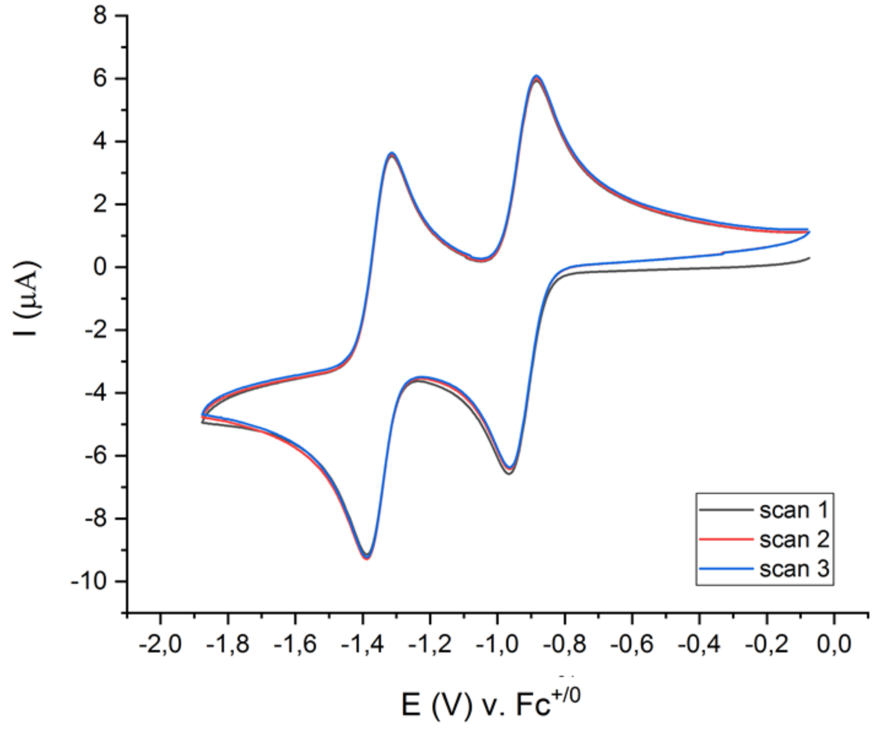

Figure S23. CV of homogeneous NDI-OMe (1mM). Supporting electrolyte: 0.5M KPF_6 in DMF. Scan rate: 50 mV s^{-1}.

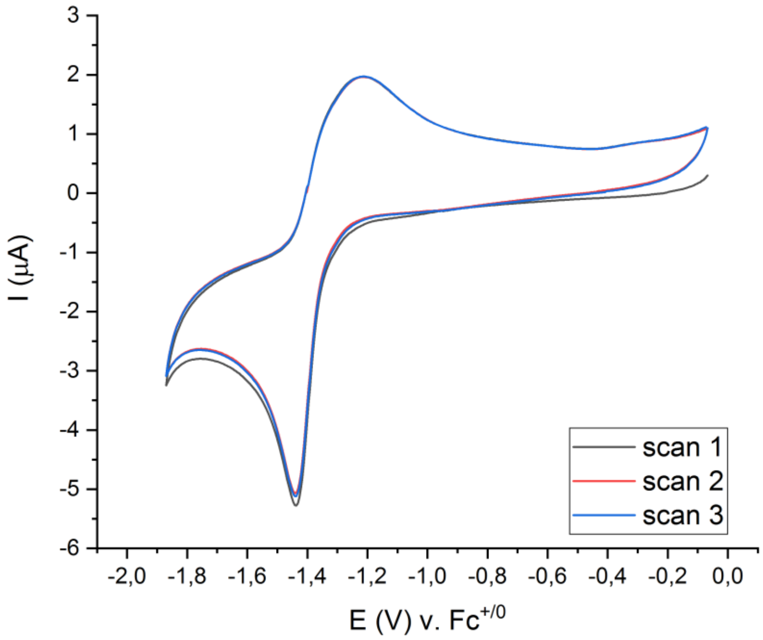

Figure S24. CV of homogeneous [FeFe](dcbdt)$(CO)_6$ (1mM). Supporting electrolyte: 0.5M KPF_6 in DMF. Scan rate: 50 mV s^{-1}.

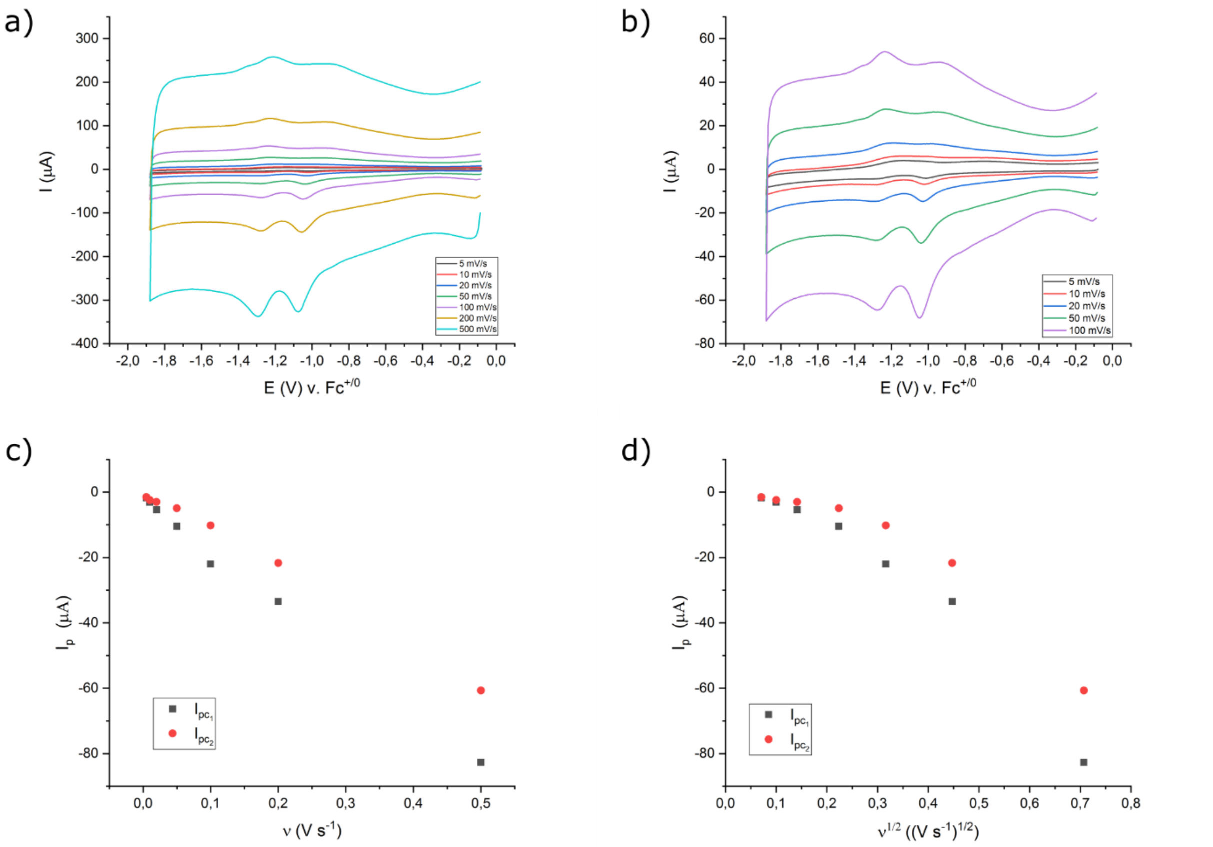

Figure S25. Scan rate analysis of PCN-700_NDI_ta. Supporting electrolyte: 0.5M KPF_6 in DMF. a) CVs of PCN-700_NDI_ta at variable scan rates from 5 mV s^{-1} through 500 mV s^{-1}. b) Closer look at CVs of PCN-700_NDI_ta at lower scan rates (5 mV s^{-1} through 100 V s^{-1}). c) Plot of cathodic peak currents for first (black) and second (red) reduction waves vs. ν. d) Plot of cathodic peak currents for first (black) and second (red) reduction waves vs. $\nu^{1/2}$.

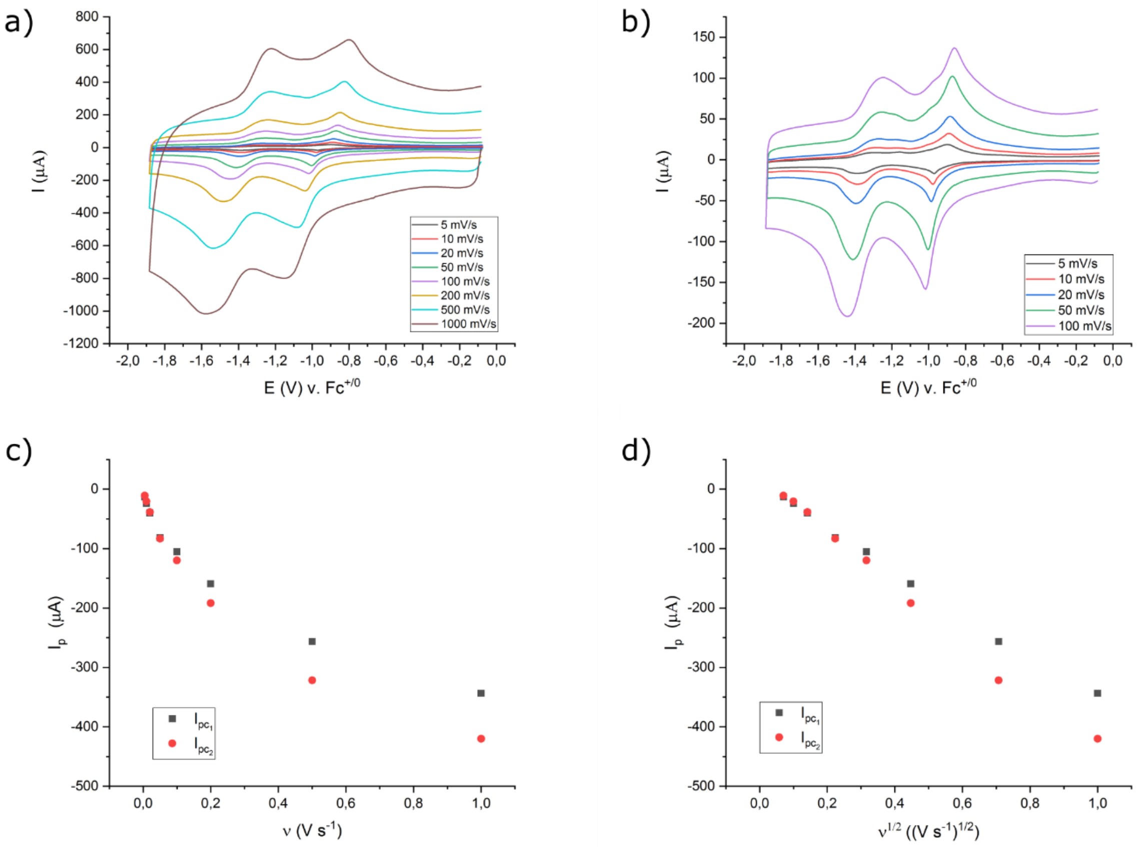

Figure S26. Scan rate analysis of PCN-700_NDI_FeFe. Supporting electrolyte: 0.5M KPF_6 in DMF. a) CVs of PCN-700_NDI_FeFe at variable scan rates from 5 mV s^{-1} through 1000 mV s^{-1}. b) Closer look at CVs of PCN-700_NDI_FeFe at lower scan rates (5 mV s^{-1} through 100 mV s^{-1}). c) Plot of cathodic peak currents for first (black) and second (red) reduction waves vs. ν. d) Plot of cathodic peak currents for first (black) and second (red) reduction waves vs. $\nu^{1/2}$.

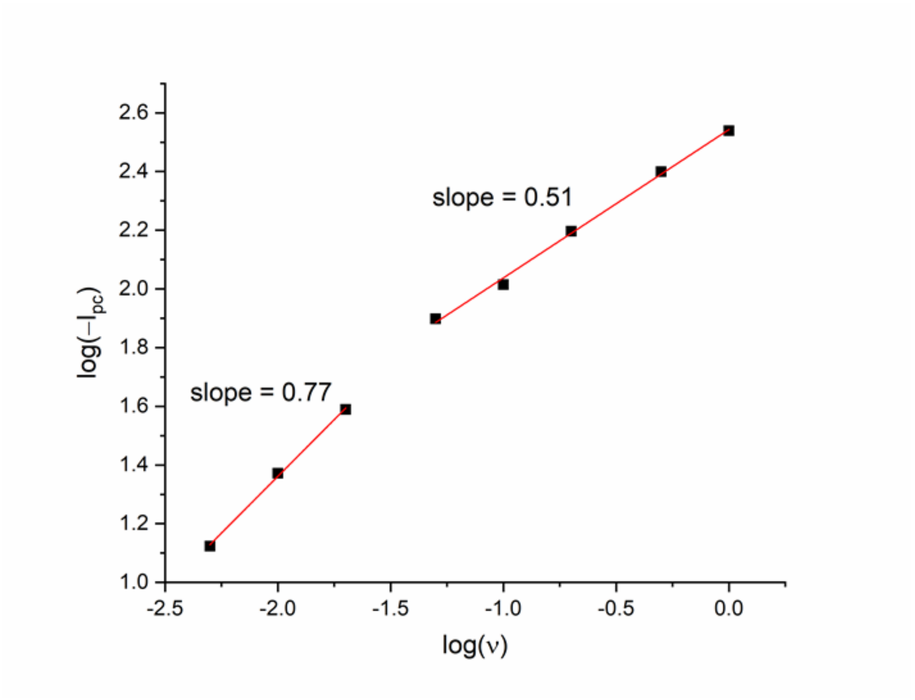

Figure S27. Double logarithmic plot of I_{pc} vs. n for the first reduction wave of PCN-700_NDI_FeFe at $E_{1/2}$ = -0.94 V.

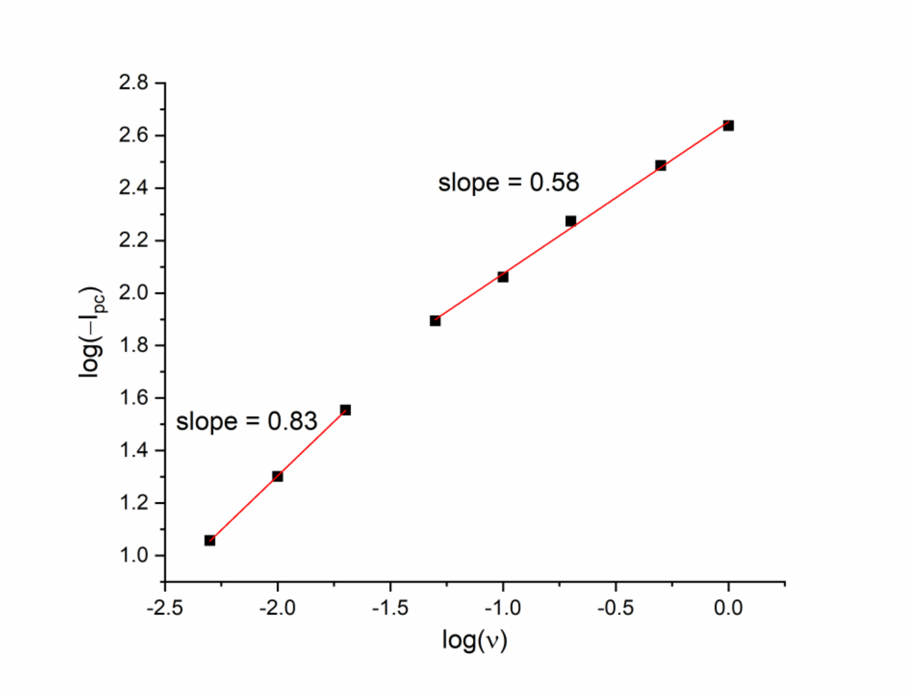

Figure S28. Double logarithmic plot of I_{pc} vs. ν for the second reduction wave of PCN-700_NDI_FeFe at $E_{1/2}$ = -1.34 V.

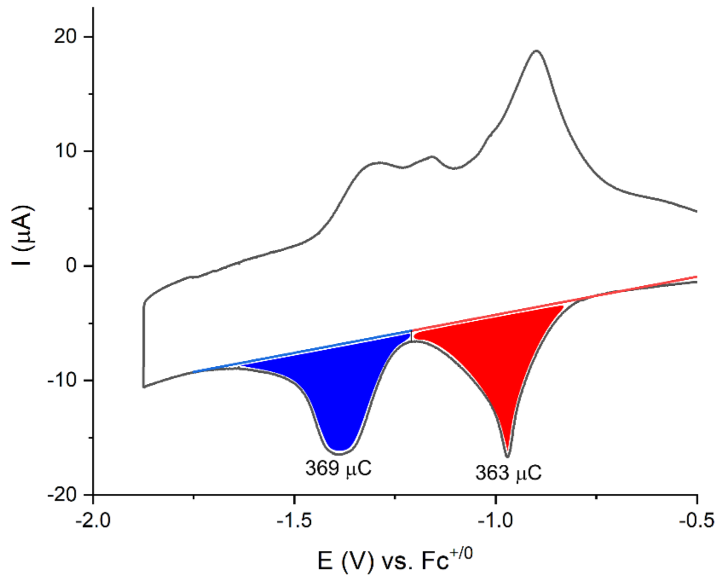

Figure S29. CV of modified MOF PCN-700_NDI_FeFe at 5 mV s^{-1} in DMF with 0.5 M KPF_6 as the supporting electrolyte. The shaded area displays the integrated current corresponding to 363 µC (red) and 369 µC (blue) for the first and second waves respectively.

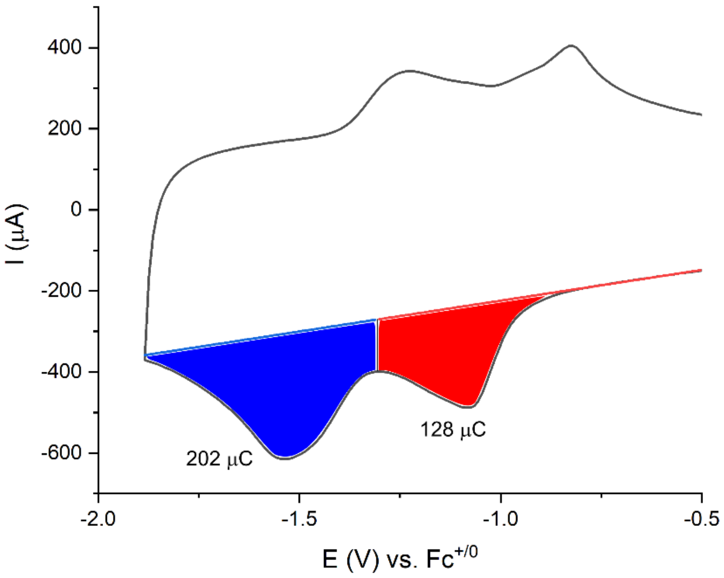

Figure S30. CV of modified MOF PCN-700_NDI_FeFe at 500 mV s^{-1} in DMF with 0.5 M KPF_6 as the supporting electrolyte. The shaded area displays the integrated current corresponding to 128 µC (red) and 202 µC (blue) for the first and second waves respectively.

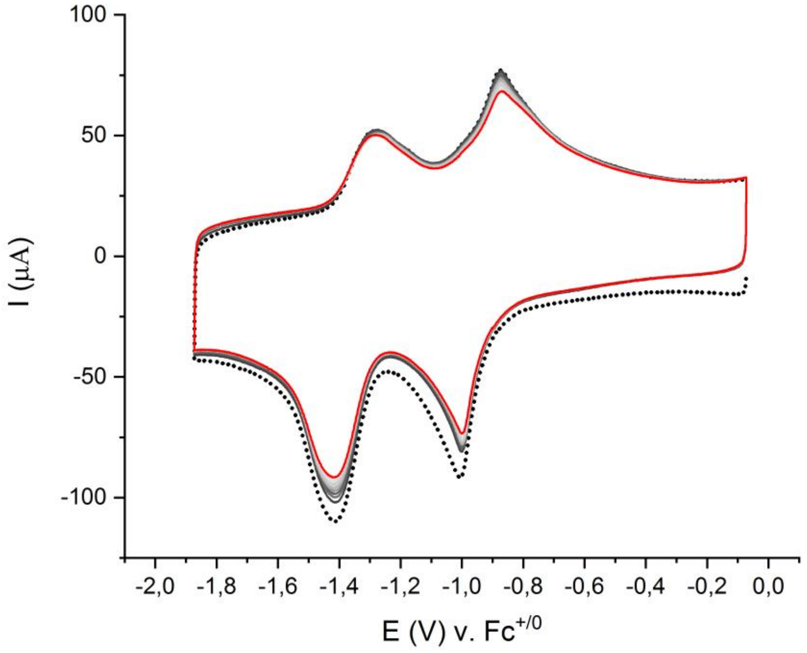

Figure S31. Stability test of PCN-700_NDI_FeFe. 10 consecutive CV scans (scan 1: dashed, scan 10: red) taken on same MOF-modified electrode after performing scan rate analysis (shown in Figure S25). Supporting electrolyte: 0.5M KPF_6 in DMF. Scan rate: 50 mV s^{-1}.

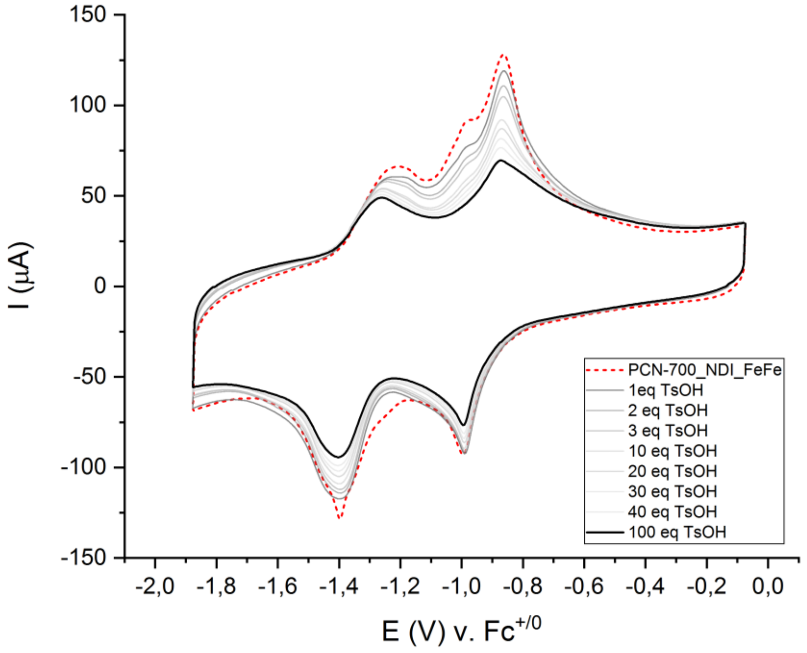

Figure S32. Tosylic acid titration to PCN-700_NDI_FeFe. No observed change in peak potential for either reductive feature due to the increasing presence of tosylic acid. Observed decrease in current likely due to delamination of MOF particles over course of the experiment. Supporting electrolyte: 0.5M KPF_6 in DMF. Scan rate: 50 mV s^{-1}.

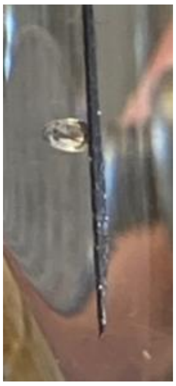

Figure S33. Electrolysis of PCN_NDI_FeFe deposited on carbon mesh electrode. Bubble formation observed during electrolysis provides visual evidence of H_2 evolution in addition to GC analysis of sampled headspace after 2 hours of electrolysis. Electrolyte: 0.1 M acetate buffer (aq) with 0.5 M KCl, pH = 5. Applied potential: -1 V (v. Ag/AgCl) for 7200 s.

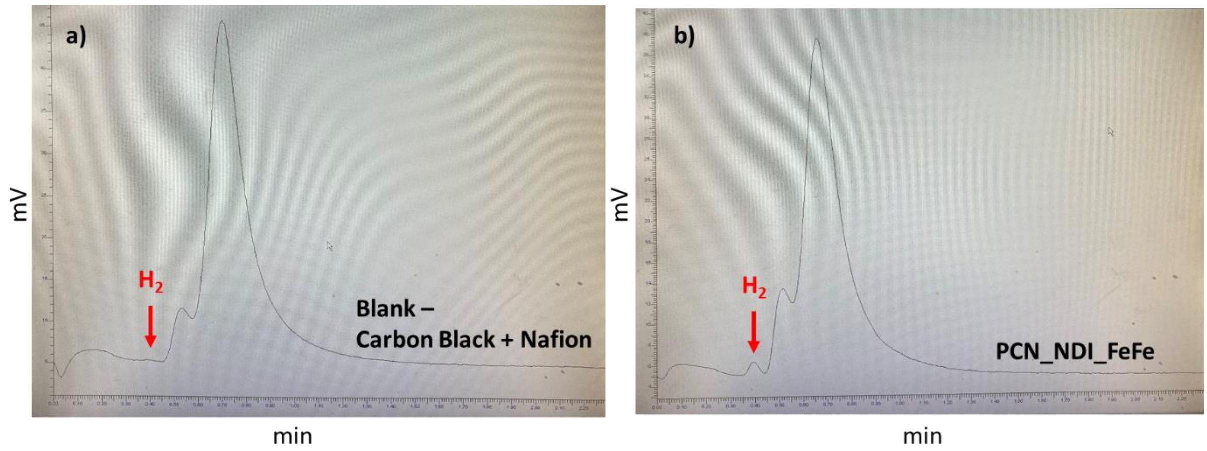

Figure S34. GC traces of sampled headspace gas (80 µL) after 2 hours of electrolysis for (a) background of binders only (carbon black + nafion) and (b) PCN-700_NDI_FeFe. H_2 was quantified by integrating the H_2 peak from the GC traces of these samples and from a linear H_2 calibration curve generated by injecting mixtures of H_2 and Ar with known H_2 content. This allowed the determination of the concentration of H_2 in the injected headspace sample. After measuring the volume of the headspace in the electrolysis cell, the total mol H_2 evolved could then be determined. This analysis gave an amount of 13 and 400 nmol H2 for the systems in a) and b), respectively.

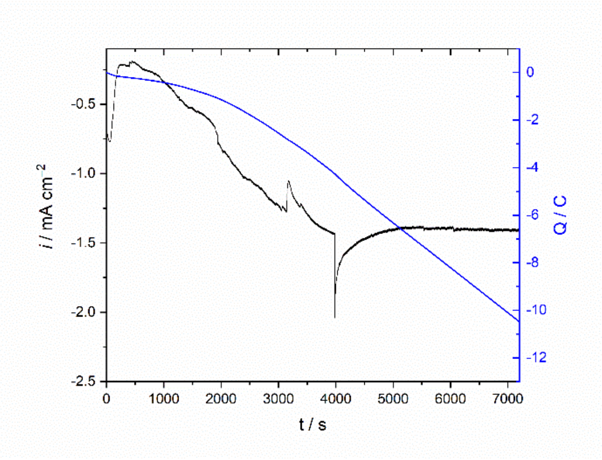

Figure S35. Controlled potential electrolysis of PCN-700_NDI_FeFe on a carbon paper electrode (carbon black + nafion) at −1V vs. Ag/AgCl in 0.1 M acetate buffer (pH = 5) with 0.5 M KCl as supporting electrolyte. Visible is a lag phase due to conditioning effects, and spikes due to bubble formation.

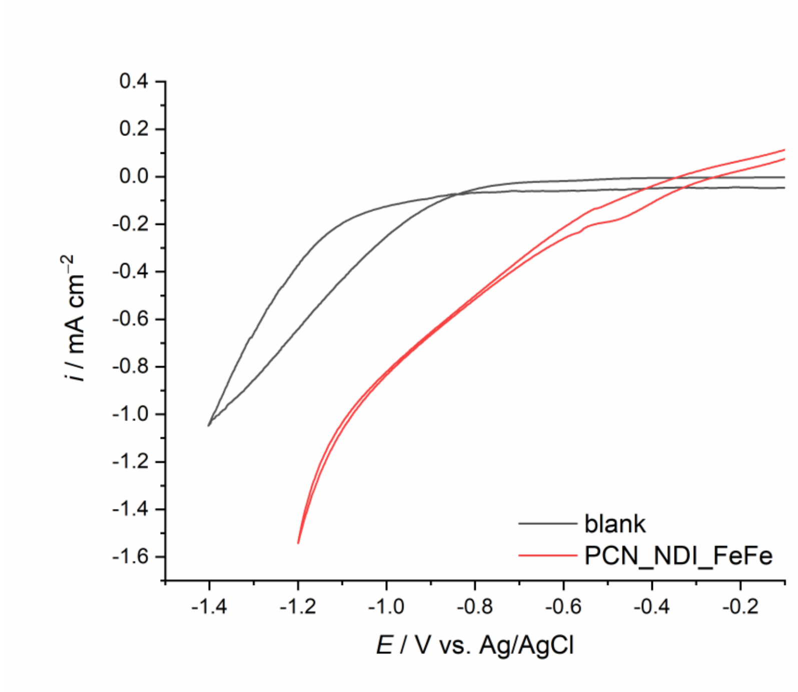

Figure S36. CVs of PCN-700_NDI_FeFe (red) on a carbon paper electrode (carbon black + nafion) in 0.1 M acetate buffer (pH = 5) with 0.5 M KCl as supporting electrolyte (recorded after 2h CPE). All scan rates were 20 mV s^{-1}. Background of binders (carbon black + nafion) on a carbon paper electrode is shown in black.

References

1. Pullen, S.; Fei, H.; Orthaber, A.; Cohen, S. M.; Ott, S., Enhanced photochemical hydrogen production by a molecular diiron catalyst incorporated into a metal-organic framework. *J. Am. Chem. Soc.* **2013,** *135* (45), 16997-17003.
2. Yuan, S.; Lu, W.; Chen, Y. P.; Zhang, Q.; Liu, T. F.; Feng, D.; Wang, X.; Qin, J.; Zhou, H. C., Sequential linker installation: precise placement of functional groups in multivariate metal-organic frameworks. *J. Am. Chem. Soc.* **2015,** *137* (9), 3177-3180.
3. Lubitz, W.; Ogata, H.; Reijerse, E.; Higuchi, Y., Chapter 11. Structure and Function of Hydrogenase Enzymes. In *Molecular Solar Fuels*, 2011; pp 288-325.
4. Li, J.; Yuan, S.; Qin, J. S.; Pang, J.; Zhang, P.; Zhang, Y.; Huang, Y.; Drake, H. F.; Liu, W. R.; Zhou, H. C., Stepwise Assembly of Turn-on Fluorescence Sensors in Multicomponent Metal-Organic Frameworks for in Vitro Cyanide Detection. *Angew. Chem. Int. Ed.* **2020,** *59* (24), 9319-9323.
5. Cychosz, K. A.; Guillet-Nicolas, R.; Garcia-Martinez, J.; Thommes, M., Recent advances in the textural characterization of hierarchically structured nanoporous materials. *Chem. Soc. Rev.* **2017,** *46* (2), 389-414.
6. Mijangos, E.; Roy, S.; Pullen, S.; Lomoth, R.; Ott, S., Evaluation of two- and three-dimensional electrode platforms for the electrochemical characterization of organometallic catalysts incorporated in non-conducting metal-organic frameworks. *Dalton Trans* **2017,** *46* (15), 4907-4911.
